# Convective stability of the critical waves of an FKPP-type model for self-organized growth

**DOI:** 10.1007/s00285-025-02189-x

**Published:** 2025-02-17

**Authors:** Florian Kreten

**Affiliations:** Institut für Angewandte Mathematik, Rheinische Friedrich-Wilhelms-Universität, Endenicher Allee 60, Bonn, 53115 Germany

**Keywords:** Traveling waves, Local stability, Convective stability, Branching particle system, Weighted space, 35B35, 35C07, 35K57, 34E10, 92C15

## Abstract

We construct the traveling wave solutions of an FKPP growth process of two densities of particles, and prove that the critical traveling waves are locally stable in a space where the perturbations can grow exponentially at the back of the wave. The considered reaction–diffusion system was introduced by Hannezo et al. (Cell 171(1):242–255, 2017) in the context of branching morphogenesis: active, branching particles accumulate inactive particles, which do not react. Thus, the system features a continuum of steady state solutions, complicating the analysis. We adopt a result by Faye and Holzer (J Differ Equ 269(9):6559–6601, 2020) for proving the stability of the critical traveling waves, and modify the semi-group estimates to spaces with unbounded weights. We use a Feynman–Kac formula to get an exponential a priori estimate for the tail of the PDE, a novel and simple approach.

## Introduction and results

We analyze an FKPP-system (Fisher 1937; Kolmogorov et al. 1937) that models a self-organized growth process. Considering the one-dimensional case $z \in \mathbb {R}, t \in \mathbb {R}^+_0$, the densities $A(t,z), I(t,z) \ge 0$ of active and inactive particles follow dynamics

1.1 $$\begin{aligned} \begin{aligned} \partial _t A&= \partial _{z}^2 A + A - A(A+I), \\ \partial _t I&= d \partial _{z}^2 I + rA + A(A+I), \qquad r,d \ge 0. \end{aligned} \end{aligned}$$

This system was introduced by Hannezo et al. (2017) in the context of branching morphogenesis. The authors used a stochastic branching particle system to model the morphogenesis of branched glandular structures. The PDE (1.1) for $d=0$ is the heuristic hydrodynamic limit of their stochastic system. Existence and uniqueness of non-negative solutions of (1.1) follow by classical fixed-point theory, see e.g. chapter 14 in Smoller (1994). Given the normalized System (1.1), the general case can be obtained by rescaling (Kreten 2022).

We construct the traveling waves of System (1.1) and prove that for $d>0$, those with minimal speed are locally stable against perturbations. The difficulty when analyzing this system is that the inactive particles *I* do not react, only the active particles *A* branch and become inactive upon collision. Thus, the system features a continuum of steady states

1.2 $$\begin{aligned} P_I = \{A = 0, I = K \, \vert \, K \in \mathbb {R}\}, \end{aligned}$$

and a priori, we do not know which of these steady states are relevant.

**Fig. 1:**
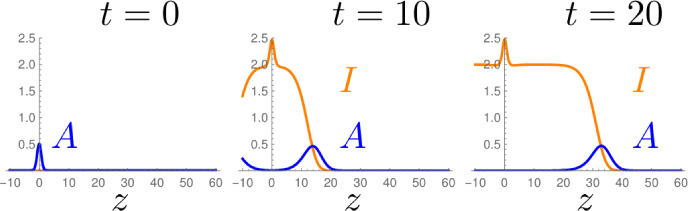
Simulation of system (1.1) for $r, d = 0$. Given an initial heap of active particles $A(z,0)=1/2 \exp (-z^2)$, and $I(z,0) = 0$, two identical traveling fronts arise, the right one is shown. After the separation of the two fronts away from the origin, the density of the remaining inactive particles is given by $I = 2$ and the front moves asymptotically with speed $c=2$

Simulations show that the system forms traveling wave solutions, which select for particular steady states among all possible ones, see Fig. 1. FKPP-systems are well-known to form heteroclinic traveling wave solutions, that connect two different steady states (Bramson 1983; Fisher 1937; Kolmogorov et al. 1937; Faye and Holzer 2020; Ducrot et al. 2019). A (right-) traveling wave solution is constant in the moving frame $$x = z -ct$$, for a wave-speed $$c >0 $$. Thereby, we refer to a traveling wave as a non-constant and bounded solution of the system of ODEs

1.3 $$\begin{aligned} \begin{aligned} 0&= c \partial _x a + \partial _{x}^2 a + a -a(a+i), \\ 0&= c \partial _x i + d \partial _{x}^2i + ra + a(a+i). \end{aligned} \end{aligned}$$

We call a solution of System (1.3) non-negative if $a,i \ge 0$. Moreover, an *invading front* is a non-negative traveling wave where both *a*(*x*) and *i*(*x*) vanish as $x \rightarrow + \infty$.

For the case $d=0$, we constructed the traveling waves of the system, but could not analyze their stability (Kreten 2022). Since the inactive particles neither react nor diffuse, any deviation from the traveling wave remains in the system for all times (see Fig. 1). Therefore, we introduce the diffusion to the inactive particles. The present article is divided into two parts:

(1) In Sect. 3, given the traveling waves for $d=0$ as our starting point, we apply perturbation techniques to construct the traveling waves for $d \ne 0$. These waves are continuous in $d \ge 0$, so we recover the original dynamics as $d \rightarrow 0$. We prove the existence of a continuum of traveling wave solutions, that correspond to the continuum of steady states (1.2), check Theorems 3.1 and 3.18. For this introduction, we restrict to the invading fronts. In particular, there exists an invading front with minimal possible speed $c=2$, referred to as *critical front*:

### Theorem 1.1

For $r \ge 0, c>0$, consider the Wave System (1.3) with

1.4 $$\begin{aligned} 0< d < \min \left\{ 1, \frac{3c}{2} , \frac{c^2}{2(r+1)} \right\} . \end{aligned}$$

If and only if $c \ge 2$, there exists an invading front. The function *i*(*x*) is decreasing, and *a*(*x*) has a unique local and global maximum. As $x \rightarrow - \infty$, the front converges exponentially fast to a fixed point $(a,i) = (0,i_{-\infty })$, where

1.5 $$\begin{aligned} 1< 2 - d \cdot \frac{ 2(r+1) }{c}< i_{-\infty }< 2. \end{aligned}$$

The rate of convergence is a function of $i_{-\infty }$, given by

1.6 $$\begin{aligned} \mu _{-\infty } = - \frac{c}{2} + \sqrt{ \frac{c^2}{4} + i_{- \infty } -1} >0. \end{aligned}$$

There are two possibilities for the speed of convergence as $x \rightarrow + \infty$. In the critical case $c = 2$, the front behaves as $x \cdot e^{-x}$. If $c>2$, convergence is purely exponential, with rate

1.7 $$\begin{aligned} \mu _{+ \infty } = \frac{c}{2} - \sqrt{ \frac{c^2}{4} -1} >0. \end{aligned}$$

Such an invading front arises in the simulation depicted in Fig. 1: a pulse of active particles is accompanied by a monotone wave of inactive particles.

(2) In Sect. 2, we analyze the stability of the critical invading front. In the moving frame $x = z -2t$, we write a solution of the PDE (1.1) as the sum of the front *a*(*x*), *i*(*x*) and the perturbations $\tilde{A}(t,x), \tilde{I}(t,x)$:

1.8 $$\begin{aligned} A(t,x ) = a(x) + \tilde{A}(t,x), \quad I(t,x) = i(x) + \tilde{I}(t,x). \end{aligned}$$

The system is not attracted towards a particular limit, as none of the Steady States (1.2) is exponentially stable as an isolated point. In principle, the system is diffusively stable at these points since all reaction-terms vanish, but controlling the nonlinear terms is difficult in a marginally stable system, where we expect only slow (algebraic) decay of the perturbations. To overcome this problematic, we operate in a space where we allow the perturbations to grow exponentially as $x \rightarrow - \infty$. This type of stability is referred to as *convective stability* (Ghazaryan et al. 2013), since we more or less ignore what happens to perturbations that are convected to the back of the invading front. To stabilize the front as $x \rightarrow + \infty$, perturbations must vanish exponentially fast, which is typical for FKPP-fronts (Sattinger 1976; Sandstede 2002). Given a smooth weight $w(x) >0$, subject to

1.9 $$\begin{aligned} w(x) = {\left\{ \begin{array}{ll} e^{- x} & \quad x \ge 1, \\ e^{ -\alpha x} & \quad x \le -1, \quad \text {with fixed } \alpha \in (0,1), \\ 1 & \quad x = 0, \end{array}\right. } \end{aligned}$$

we prove that if the weighted perturbations

1.10 $$\begin{aligned} \frac{\tilde{A}(t,x)}{w(x)}, \quad \frac{ \tilde{I}(t,x)}{w(x)} \end{aligned}$$

are initially small, they vanish point-wise with algebraic decay $t^{-3/2}$. In order to deal with the unbounded weight, we need some a priori control of the left tail of the PDE. As a novelty, we present an easy-to-use a priori estimate. In the moving frame, we estimate the decay of a linear super-solution via a Feynman–Kac formula, where we can stop the underlying shifted Brownian motion at any point that suits our needs. The resulting estimate is based on an easy intuition, and can readily be adapted to other systems under three conditions: First, the system is asymptotically monotone around the investigated steady state. Second, the diffusion is not too large, such that the dynamics of the system are dominated by its reaction. Third, the critical components of the system that drive the reaction decay exponentially in the chosen regime. A mathematical review of related works is given in Sect. 4.

We need to assume that the discrete spectrum of the linearized perturbation equation is stable, check Sect. 2.3 for the precise statement. We verify this numerically, the technique is presented in Appendix B.

### Theorem 1.2

For a pair $d >0, r \ge 0$ as in Theorem 1.1, consider the critical invading front with speed $c=2$. If we assume that the discrete spectrum of the weighted linearized perturbation equation contains no elements with non-negative real-part (see 2.3), then the critical invading front is locally stable in a space with weight *w*(*x*) as above (1.9).

Fix a pair of constants $C, \mu _0 >0$. For all $\epsilon >0$, there exists a $\delta >0$, such that if the unweighted perturbations fulfill

1.11 $$\begin{aligned}&(i)&\forall x \le 0: \quad \vert \tilde{A}(0,x) \vert \le C e^{\mu _0 x}, \quad \vert \tilde{I}(0,x)&\vert \le \delta , \end{aligned}$$

and if the weighted perturbations $\tilde{A}(0,x)/w(x), \tilde{I}(0,x) / w(x)$ are uniformly continuous up to the second derivative, and

1.12 $$\begin{aligned}&(ii)&\int _{ \mathbb {R}}(1+ \vert x \vert ) \Bigg [ \Big \vert \frac{\tilde{A}(0,x)}{w(x)} \Big \vert + \Big \vert \frac{ \tilde{I}(0,x)}{w(x)} \Big \vert \Bigg ]\, dx&\le \delta , \end{aligned}$$

1.13 $$\begin{aligned}&(iii)&\left\| \frac{\tilde{A}(0,x)}{w(x)} \right\| _{\infty } + \left\| \frac{ \tilde{I}(0,x)}{w(x)} \right\| _{\infty }&\le \delta , \end{aligned}$$

then the weighted perturbations decay point-wise with algebraic speed $t^{3/2}$:

1.14 $$\begin{aligned} \sup _{t \ge 0} \sup _{x \in \mathbb {R}} \, \frac{(1+t)^{3/2}}{w(x) (1+ \vert x\vert ) } \Big ( \vert \tilde{A}(t,x) \vert + \vert \tilde{I}(t,x) \vert \Big ) \le \epsilon . \end{aligned}$$

### Biological background

In a *self-organized* system, the emergence of a macroscopic structure is determined solely by the behavior of its microscopic components. These components interact with and react to their local environment, without any additional global guiding mechanism. Biology abounds with phenomena that can be explained (at least partially) via self-organization of groups of individuals: Bacterial populations express pattern formation depending on their environment (Keller and Segel 1971; Painter 2019), ant colonies are able to build nests, and corals and sponges which are built of repeated small units, form complex branched organisms (Kaandorp 2001). In particular, cellular systems constitute a huge playground for possible self-organization phenomena and are able to express all different kinds of behavior: spontaneous pattern formation, traveling waves, oscillations and metastable states with fast switching (Karsenti 2008; Misteli 2001; Wedlich-Söldner and Betz 2018). It is believed that self-organization is vital for the robustness and the adaptability of cellular organization and the maintenance of tissues in general, though many of the underlying mechanisms are still unexplored (Hannezo and Simons 2018; Wedlich-Söldner and Betz 2018).

Hannezo et al. (2017) proposed “A Unifying Theory of Branching Morphogenesis”. The aim of this study was to find a set of coherent microscopic rules that can explain the growth of the branched structures on the macroscopic scale. The authors modeled the branching morphogenesis of glandular structures in organs via a stochastic system that is based on branching and annihilating random walks (Cardy and Täuber 1996). In this model, a branched structure is represented by a network that undergoes stochastic growth dynamics. The proposed algorithm is based on simple local mechanisms, that generate a randomly growing binary tree which fills a given volume (e.g. open, bounded sets in $\mathbb {R}^2$ or $\mathbb {R}^3$). Essentially, there are two types of mechanisms: at its tip, each branch of the tree elongates or splits up at certain rates, and these tips are called *active*. However, when an active tip comes too close to a different branch, it irreversibly ceases any activity and becomes inactive. Thus, the main part of the network is entirely static, consisting of inactive branches. Rather surprisingly, the interior of the network exhibits a homogeneous geometry, in particular a constant density of branches, while the active tips concentrate at the boundary and form a sharp layer of growth. This self-organization is quite remarkable, but lacks any mathematical explanation so far. Since the active tips can interact with their past trajectories, the system is not Markovian, which highly complicates any rigorous analysis. A similar problem arises when analyzing the stability of the critical wave for $d=0$.

To somehow study their model analytically, Hannezo et al. suggested the PDE (1.1) with $d=0$, which is the heuristic mean-field limit of the stochastic system. They present only a numerical analysis, but could predict some of our results. Simulations indicate that System (1.1) forms traveling solutions for a wide range of initial data, an example is depicted in Fig. 1. Among those traveling waves, those with minimal speed are of particular relevance, as they seem to arise in the large time limit for any compact initial data. These waves with minimal speed are referred to as *critical*, we present the mathematical background in Sect. 4.

We construct these critical traveling wave solutions, and prove their stability against small perturbations. Even though it is only a first step into this direction, this paper sheds light at the ability of growth-processes related to (1.1) to self-organize, and at the robustness of this mechanism. In particular, our findings strengthen the observation that the stochastic system proposed by Hannezo et al. (2017) reliably generates a particular class of branched structures in a self-organized fashion, and that this process is robust against small errors of individual cells. The underlying assumption of a logistic growth is natural, so similar rules might drive and regulate other growth-processes as well, without relying on global guiding gradients.

### Outline of the paper

In Sect. 2, we prove the local stability of the critical invading front, which is the novelty of the present work. After introducing the necessary objects in Sect. 2.1, we present the central steps of the proof in Sect. 2.2. The technical details are then given in Sect. 2.3 and Sect. 2.4. In Appendix A, we present the (standard) computation of the essential spectrum of the linearized perturbation, in Appendix B, we present a numerical evaluation of the non-negative discrete spectrum. The Feynman–Kac estimate, which controls the left tail of the PDE, is proven in Appendix C.

In Sect. 3, we construct the traveling waves. Using the existing traveling waves for $d=0$ (Thm. 3.1) as a starting point, we apply perturbation techniques to track any finite segment of the waves for $d \ne 0$, see Sect. 3.2. The singular perturbation (for passing continuously from $d=0$ to $d \sim 0$) is explained in Appendix D. In Sect. 3.3, we analyze the phase space of the non-negative waves. We then extend the previous perturbation result and prove that a traveling wave persists locally under perturbation in *d*, up to its limits. We prove an estimate of type $i_{-\infty }+ i_{+\infty }= 2 + \mathcal {O}(d)$ regarding the limits of a traveling wave in Sect. 3.4. We then prove the existence of non-negative traveling waves and invading fronts up to $d \sim 1$, in Sect. 3.5.

A mathematical outlook at FKPP systems and related stability results is presented in Sect. 4.

## The stability of the critical invading front

### Notation

In the following, we assume without further mentioning that *a*(*x*), *i*(*x*) is a non-negative critical invading front as in Theorem 1.1. To begin with, we decompose any solution of the PDE (1.1) in the moving frame $x = z -ct$ into

2.1 $$\begin{aligned} A(t,x) = a(x) + \tilde{A}(t,x), \quad I(t,x) = i(x) + \tilde{I}(t,x). \end{aligned}$$

Then, the perturbations $\tilde{A}(t,x)$ and $\tilde{I}(t,x)$ follow

2.2 $$\begin{aligned} \begin{aligned} \partial _t \tilde{A}&= \partial _{x}^2 \tilde{A} + c \partial _x\tilde{A} + \tilde{A}(1-2a-i) - \tilde{I}a - \tilde{A}(\tilde{A} + \tilde{I}),\\ \partial _t\tilde{I}&= d \partial _{x}^2 \tilde{I} + c \partial _x \tilde{I} + \tilde{A}(2a+i+r) + \tilde{I}a +\tilde{A}(\tilde{A} + \tilde{I}). \end{aligned} \end{aligned}$$

For $\tilde{A},\tilde{I}$ that solve the perturbation Eq. (2.2) and given a strictly positive weight *w*(*x*), we define the weighted perturbations $u: = \tilde{A} / w, v: = \tilde{I} /w$. If *w* is twice differentiable with derivatives $w',w''$, they solve

2.3 $$\begin{aligned} \begin{aligned} \partial _t u&= \partial _{x}^2 u + \partial _x u \cdot \bigg ( c + 2\frac{w'}{w} \bigg ) + u \cdot \bigg ( c \frac{w'}{w} + \frac{ w''}{w} \bigg ) \\&\quad + u( 1-2a-i ) - va -wu(u+v), \\ \partial _t v&= d \partial _{x}^2 v + \partial _x v \cdot \bigg ( c + 2d\frac{w'}{w} \bigg ) + v \cdot \bigg ( c \frac{w'}{w} + d \frac{ w''}{w} \bigg ) \\&\quad + u( 2a+i+r) +va +wu(u+v). \end{aligned} \end{aligned}$$

Splitting this into linear and nonlinear part:

2.4 $$\begin{aligned} \partial _t \begin{pmatrix} u \\ v \end{pmatrix}&= \mathcal {L} \begin{pmatrix} u \\ v \end{pmatrix} + \mathcal {N}\begin{pmatrix} u \\ v \end{pmatrix}, \end{aligned}$$

we write the linear part as

2.5 $$\begin{aligned} \mathcal {L} = \begin{pmatrix} \mathcal {L}_u & \mathcal {L}_{12} \\ \mathcal {L}_{21} & \mathcal {L}_v \end{pmatrix} \end{aligned}$$

with

2.6 $$\begin{aligned} \mathcal {L}_u&:= \partial _{x}^2 + \left( c + 2\frac{w'}{w}\right) \partial _x + 1-2a-i + c \frac{w'}{w} + \frac{ w''}{w} \end{aligned}$$

2.7 $$\begin{aligned} \mathcal {L}_{12}&:= -a \end{aligned}$$

2.8 $$\begin{aligned} \mathcal {L}_v&:= d \partial _{x}^2 + \left( c + 2d\frac{w'}{w} \right) \partial _x + a+ c \frac{w'}{w} + d \frac{ w''}{w} \end{aligned}$$

2.9 $$\begin{aligned} \mathcal {L}_{21}&:= 2a+i+r \end{aligned}$$

and the nonlinear part

2.10 $$\begin{aligned} \mathcal {N} \begin{pmatrix} u \\ v \end{pmatrix}&:= \begin{pmatrix} -wu(u+v) \\ wu(u+v) \end{pmatrix}. \end{aligned}$$

Lastly, we define as $\mathcal {G}(t,x,y)$ the Kernel of the linear Eq. (2.5):

2.11 $$\begin{aligned} \mathcal {G}(t,x,y) := \begin{pmatrix} \mathcal {G}_{11}(t,x,y) & \mathcal {G}_{12}(t,x,y) \\ \mathcal {G}_{21}(t,x,y) & \mathcal {G}_{22}(t,x,y) \end{pmatrix}, \end{aligned}$$

acting on a space to be defined later. We summarize the weighted perturbations as $$p = \begin{pmatrix}u \\ v \end{pmatrix}$$. In this compact notation, we will estimate the evolution of the weighted perturbations *p*(*t*, *x*) with a Duhamel principle:

2.12 $$\begin{aligned} p (t,x) = \int _{ \mathbb {R}} \mathcal {G}(t,x,y) p (0,y) \, dy + \int _0^t ds \int _\mathbb {R} \mathcal {G}(t-s,x,y) \mathcal {N} (p)(s,y) \, dy. \end{aligned}$$

### Central steps

For $d>0$ and in a function space where the weight grows exponentially as $x \rightarrow - \infty$, the operator $\mathcal {L}$ is sectorial (Henry 1981; Alexander et al. 1990), see Sect. 2.3 for an analysis of its spectrum: the spectrum is contained in the strictly negative half-plane, with the exception of a single half-line that touches the origin. In this setting, we can use a result of Faye and Holzer (2020) to estimate the long-time behavior of $\mathcal {G}$, check Sect. 2.4. Roughly, this result states that $\mathcal {G}$ decays like $t^{-3/2}$ for large *t*. Then, for estimating the evolution of the full nonlinear system via Eq. (2.12), it is crucial to control the nonlinear integral

2.13 $$\begin{aligned} \int _{\mathbb {R}} w (x) u(t,x) \cdot \big ( u(t,x) + v(t,x) \big ) \, dx . \end{aligned}$$

We treat the cases $x \le 0$ and $x \ge 0$ separately. Assuming that *w*(*x*) vanishes exponentially fast as $z \rightarrow + \infty$, the front satisfies the classical estimate

2.14 $$\begin{aligned} \begin{aligned}&\Big \vert \int _{0}^\infty w (x) u(t,x) \cdot \big ( u(t,x) + v(t,x) \big ) \, dx \Big \vert \\&\quad \le \sup _{x \ge 0} \Big \{ \big ( \vert u(t,x) \vert + \vert v(t,x) \vert \big )^2 \Big \} \cdot \int _{0}^\infty w (x) \, dx\\&\quad \le C \cdot \sup _{x \ge 0} \Big \{ \big ( \vert u(t,x) \vert + \vert v(t,x) \vert \big )^2 \Big \}. \end{aligned} \end{aligned}$$

Since *w*(*x*) grows exponentially as $x \rightarrow - \infty$, we take a different approach for $x \le 0$. This is where we need the a priori estimate:

#### Proposition 2.1

Let *A*(*t*, *x*), *I*(*t*, *x*) be a non-negative solution of the PDE (1.1) in the moving frame $x = z -ct$, for a speed $c > 0$. Assume that there exist constants $K, \delta , \mu _0 >0$, such that the initial data fulfill

2.15 $$\begin{aligned} I(0, x)&\ge 1 + \delta &  \qquad \forall x \le 0, \nonumber \\ A(0,x)&\le K e^{\mu _0 x} &  \qquad \forall x \le 0, \nonumber \\ A(0,x) + I(0,x)&\le K &  \qquad \forall x \in \mathbb {R}. \end{aligned}$$

Moreover, assume that for some time $t \in (0, \infty ]$, it holds that

2.16 $$\begin{aligned} I(s, x = 0)&\ge 1 + \delta &  \qquad \forall s \in [0,t). \end{aligned}$$

Then, there exist $C, \zeta >0$ that are independent of *t*, such that:

2.17 $$\begin{aligned} \forall s \in [0,t), \, x \le 0: \quad&i)&\quad I(s,x) \ge 1 + \delta , \end{aligned}$$

2.18 $$\begin{aligned}&ii)&\quad A(s,x) \le C e^{\zeta x}. \end{aligned}$$

Essentially, the inequality $I \ge 1 + \delta$ implies the exponential decay of *A*, the speed of decay $\zeta$ is thus of order $\delta$. The proof and the used Feynman–Kac formula are standard, given in Appendix C. The way we apply this result for controlling the perturbations is new. For the wave it holds that $\lim _{x \rightarrow - \infty }i(x) >1$, where *i*(*x*) is monotone. We first shift the wave such that $i(0) = 1 + 2 \delta > 1$. Then, it suffices to control

2.19 $$\begin{aligned} \vert \tilde{I}(t,x=0) \vert \le \delta \qquad \forall t \ge 0, \end{aligned}$$

and in view of Proposition 2.1, there exist constants $C, \zeta >0$ such that

2.20 $$\begin{aligned} \vert \tilde{A}(t,x) \vert \le A(t,x)&\le C e^{\zeta x} \qquad \forall x \le 0, \, t \ge 0, \end{aligned}$$

since $A \ge 0$. We re-substitute $w u = \tilde{A}$ to estimate

2.21 $$\begin{aligned} \begin{aligned}&\Big \vert \int _{-\infty }^0 w (x) u(t,x) \cdot \big ( u(t,x) + v(t,x) \big ) \Big \vert \, dx\\&\quad \le \int _{-\infty }^0 \vert \tilde{A}(t,x) \vert \cdot \vert u(t,x) + v(t,x) \vert \, dx\\&\quad \le \sup _{x \le 0} \Big \{ \vert u(t,x) \vert + \vert v(t,x) \vert \Big \} \cdot \int _{-\infty }^0 \vert \tilde{A}(t,x) \vert \, dx\\&\quad \le C \cdot \sup _{x \le 0} \Big \{ \vert u(t,x) \vert + \vert v(t,x) \vert \Big \}. \end{aligned} \end{aligned}$$

Given both Estimates (2.14) and (2.21), the resulting semi-group estimates are quite standard, though a bit lengthy due to the different cases that need to be dealt with, presented in Sect. 2.4.

### An appropriate function space

Here and in the following, let $\mathcal {L}$ be defined by (2.5), as an operator

2.22 $$\begin{aligned} \mathcal {L}: \mathcal {H}^2(\mathbb {R}) \times \mathcal {H}^2(\mathbb {R}) \rightarrow L^2(\mathbb {R}) \times L^2(\mathbb {R}), \end{aligned}$$

where $\mathcal {H}^2$ is the $L^2$ Sobolev space, dense in $L^2$. Given a pair of exponents $\alpha _\pm >0$, we fix a smooth weight $w(x) >0$, subject to the conditions

2.23 $$\begin{aligned} w(x) := {\left\{ \begin{array}{ll} e^{- \alpha _+ x} & \quad x \ge 1, \\ e^{- \alpha _- x} & \quad x \le -1, \\ 1 & \quad x = 0. \end{array}\right. } \end{aligned}$$

Note that this weight is bounded for $x \ge 0$, and unbounded for $x \le 0$. The choice of the weight *w* has a critical influence on the spectrum of $\mathcal {L}$. We want to emphasize that for a critical front, there is only one possible choice for $\alpha _+$, whereas $\alpha _- >0$ is necessary for considering the convective regime.

**Fig. 2:**
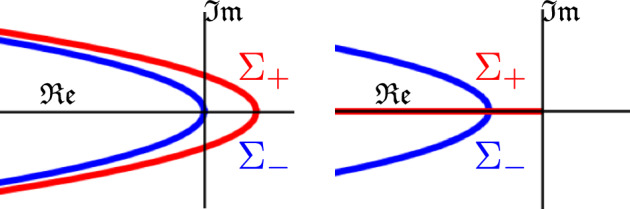
The two critical parts $\Sigma _+$ and $\Sigma _-$ of the essential spectrum of $\mathcal {L}$ as in Proposition 2.2, defined by (2.25) and (2.28). On the left, the essential spectrum is unstable in an unweighted space. On the right, the essential spectrum is stabilized via an exponential weight, with Exponents (2.24). The line $\Sigma _+$ implies that $\mathcal {L}$ is at most marginally stable, with essential spectrum up to the origin

#### Proposition 2.2

For $d \in (0,1)$ and exponents

2.24 $$\begin{aligned} \alpha _- \in ( 0, 1), \quad \alpha _+ = 1, \end{aligned}$$

consider an exponential weight *w*(*x*) as in (2.23). Given a critical invading front, consider the linear part $\mathcal {L}$ of the perturbations in the moving frame (2.5), defined on spaces (2.22). Then, the Fredholm borders, which bound the essential spectrum of $\mathcal {L}$, consist of the union of three parabolas

2.25 $$\begin{aligned} \Sigma _-&:= \Big \{ \lambda \in \mathbb {C} \big \vert \, \mathfrak {Re}\lambda = \frac{1}{d} \big ( 2(1-d\alpha _-)^2 - 1 - \vert \lambda +1 \vert \big ) \Big \}, \end{aligned}$$

2.26 $$\begin{aligned} \Sigma _2&:= \Big \{ \lambda \in \mathbb {C} \big \vert \, \mathfrak {Re}\lambda = \frac{1}{d} \big ( 2 (1-d)^2 -1 - \vert d\lambda +1 \vert ^2 \big ) \Big \}, \end{aligned}$$

2.27 $$\begin{aligned} \Sigma _3&:= \Big \{ \lambda \in \mathbb {C} \big \vert \, \mathfrak {Re}\lambda = - i_{-\infty }- 2(1-\alpha _-)^2 - \vert i_{-\infty }+ \lambda \vert \Big \}. \end{aligned}$$

that lie in the strict negative half-plane, and the marginally stable half-line

2.28 $$\begin{aligned} \Sigma _{+}&= \big \{ \lambda \in \mathbb {C} \big \vert \, \mathfrak {Re}\lambda \le 0, \mathfrak {Im} \lambda = 0 \big \}. \end{aligned}$$

The proof of Proposition 2.2 is standard, we present it in Appendix A for completeness. The two critical parts $\Sigma _+$ and $\Sigma _-$ of the essential spectrum are depicted in Fig. 2. This type of essential spectrum is identical to that in Faye and Holzer (2020). So far, we could not analyze the discrete spectrum of $\mathcal {L}$ analytically, and make the following

#### Assumption 2.3

(*No resonant zero, no unstable point spectrum*) In the setting of the above Proposition 2.2, let $\Sigma _{d}$ be the discrete spectrum of the weighted linear operator $\mathcal {L}: \mathcal {H}^2 \rightarrow L^2$. Assume that $\Sigma _d$ contains no elements with non-negative real-part, and moreover, assume that there is no bounded solution to the equation $\mathcal {L}u=0$.

We verify this assumption numerically, check Appendix B. Note that for an invading front *a*(*x*), *i*(*x*), it is easy to see that the symbolic equation $\mathcal {L}(a',i') = 0$ holds. However, the critical front behaves like $x e^{-x}$ as $x \rightarrow + \infty$, so its derivative is not an element of the considered weighted space. If there would be a bounded solution of $\mathcal {L}u=0$, one would refer to this as a resonance of $\mathcal {L}$.

### Estimating the long-time behavior

We prove the stability of the critical invading front using a linear stability result by Faye and Holzer (2020), which—to our knowledge—was the first the stability result for a critical pulled front with more than one reactant. This result is not system-specific (even if the authors do not mention it explicitly), with the only limitation that it is restricted to the case of two reactants, and diffusive sectorial operators with shape of the spectrum as described above, see Proposition 2.2 and Assumption 2.3.

A more general, and slightly stronger statement which we could also apply is given by Avery (2024). Still, we use the result in Faye and Holzer (2020), since the proof is almost self-contained and presented in a very reader-friendly way. The two works Faye and Holzer (2020) and Avery (2024) essentially follow a similar approach: they estimate the temporal Green’s function via a sectorial contour integral that approaches the origin as close as possible. We refer to Sections 2–4 in Faye and Holzer (2020) for a compact presentation of the details. Likewise, we refer to Proposition 5.5 in Avery (2024), many of the underlying calculations are presented in Avery and Scheel (2021), Avery and Scheel (2022):

#### Theorem 2.4

(cf. Prop. 4.1 and Lemma 5.1 in Faye and Holzer (2020), see also Prop. 5.5 in Avery (2024)) For $\mathcal {L}$ with essential spectrum as in Proposition 2.2, and given that the discrete spectrum of $\mathcal {L}$ fulfills Assumption 2.3, the temporal Green’s function $\mathcal {G}(t,x,y)$ for $\partial _t (u,v) = \mathcal {L}(u,v)$ satisfies the following estimates: there exists constants $C, \kappa > 0$, such that for all pairs of indices $i,j \in \{1,2\}$ and for all $x,y \in \mathbb {R}$:

2.29 $$\begin{aligned}&\big \vert \mathcal {G}_{ij}(t,x,y) \big \vert \le C \frac{1}{t^{1/2}} e^{- \frac{ \vert x-y \vert ^2}{\kappa t} } &  \quad \forall t < 1, \end{aligned}$$

2.30 $$\begin{aligned}&\int _{\mathbb {R}} \big \vert \mathcal {G}_{ij}(t,x,y) \big \vert \cdot \vert h(y) \vert \, dy \le C \cdot \vert \vert h \vert \vert _\infty &  \quad \forall t < 1. \end{aligned}$$

Moreover, for all $t\ge 1$ and $x \in \mathbb {R}$:

2.31 $$\begin{aligned} \int _{\mathbb {R}} \big \vert \mathcal {G}_{i,j}(t,x,y) \big \vert \cdot \vert h(y) \vert \, dy \le C \cdot \frac{1 + \vert x \vert }{(1+t)^{3/2}} \int _\mathbb {R} (1+ \vert y \vert ) \cdot \vert h(y) \vert \, dy. \end{aligned}$$

In Faye and Holzer (2020), the authors consider an integrable weight *w*(*x*) and follow the reasoning behind Inequality (2.14). We extend their proof by using the a priori bounds that we have for the left tail of the system, see (2.21). The following standard Lemma helps controlling the resulting integrals:

#### Lemma 2.5

(Lemma 2.3 in Xin (1992)) Let $\alpha , \beta , \gamma ,t >0$ with $\alpha \le \beta + \gamma -1$. If either $\alpha \le \beta , \gamma \ne 1$, or $\alpha < \beta , \gamma = 1$, then there exists a constant *C* such that

2.32 $$\begin{aligned} \int _0^{\frac{t}{2}} \frac{1}{(1+t-s)^\beta } \frac{1}{(1+s)^\gamma } \, ds&\le C \frac{1}{(1+t)^\alpha }. \end{aligned}$$

Similarly, if either $\alpha \le \gamma , \beta \ne 1$, or $\alpha < \gamma , \beta = 1$, then

2.33 $$\begin{aligned} \int _{\frac{t}{2}}^t \frac{1}{(1+t-s)^\beta } \frac{1}{(1+s)^\gamma } \, ds&\le C \frac{1}{(1+t)^\alpha }. \end{aligned}$$

We can now prove the stability of the critical front:

#### Proof of Theorem 1.2

We adopt the notation introduced in Sect. 2.1. That is, we consider vector $p = (u,v)$ of the weighted perturbations, and moreover write $\vert p(t,x) \vert = \vert u (t,x) \vert + \vert v(t,x) \vert$. Note that since $\vert wu \vert = \vert \tilde{A} \vert \le 1$, all reaction-terms of the perturbation System (2.3) are at most linear in $\vert p \vert$. Thus, by a standard Gronwall estimate and a fixed-point argument, a unique solution of System (2.3) exists in *BUC* for arbitrarily long times, check e.g. chapter 14 in Smoller (1994), and Thm. 46.4 and concluding remarks in Sell and You (2002).

We will prove that

2.34 $$\begin{aligned} \Theta (t) := \sup _{s \le t} \sup _{x \in \mathbb {R}} \frac{(1+s)^{3/2}}{1+ \vert x \vert } \vert p(s,x) \vert \end{aligned}$$

is bounded uniformly in $t \ge 0$, if the initial data is small enough. We introduce a border $B \le 0$ to be specified later, and split the Duhamel Formula (2.12):

2.35 $$\begin{aligned} \vert p(t,x) \vert&\le \Big \vert \int _{\mathbb {R}} \mathcal {G}(t,x,y) p(0,y) \, dy \Big \vert \end{aligned}$$

2.36 $$\begin{aligned}&\quad + \Big \vert \int _0^t ds \int _{-\infty }^B \mathcal {G}(t-s,x,y) \mathcal {N}(p)(s,y) \, dy \Big \vert \end{aligned}$$

2.37 $$\begin{aligned}&\quad + \Big \vert \int _0^t ds \int _{B}^{+\infty } \mathcal {G}(t-s,x,y) \mathcal {N}\big (p)(s,y) \, dy \Big \vert . \end{aligned}$$

For $y \ge B$, we use the exponential decay of *w*(*y*), whereas for $y \le B$, we use the exponential decay of *A*(*t*, *y*), see (2.21). Theorem 2.4 yields different results for $\mathcal {G}$ for $t \le 1$ and $t \ge 1$, thus we also differentiate the above terms for $t-s \le 1$ and $t-s \ge 1$. In the following, we let *C* be a universal constant that does not depend on *B*, whereas $C_B$ will be a universal constant that does. The constant $C_B$ will become quite large, but will be the prefactor of an estimate of order $\Theta ^2$, whereas we choose *B* such we can control any term that is linear in $\Theta$.

We can shift the critical front *a*(*x*), *i*(*x*) such that there exists a $\delta _I >0$ with

2.38 $$\begin{aligned} i(x) \ge 1 + 2 \delta _I \qquad \forall x \le 0. \end{aligned}$$

We assume that the initial perturbations are small: $\vert \vert \tilde{I}(0,\cdot ) \vert \vert _\infty \le \delta _I$, then

2.39 $$\begin{aligned} I(0,x) = i(x) + \tilde{I}(0,x) \ge 1 + \delta _I \qquad \forall x \le 0. \end{aligned}$$

For the moment, we also assume that

2.40 $$\begin{aligned} \vert \tilde{I}(t,x=0)\vert = \vert v(t,x=0) \vert \le \delta _I \qquad \forall t \ge 0, \end{aligned}$$

which in particular implies that

2.41 $$\begin{aligned} I(t,x=0) \ge 1 + \delta _I \qquad \forall t \ge 0. \end{aligned}$$

We will verify (2.40) a posteriori, by proving that the perturbations stay small. Given (2.39) and (2.40), we can apply Proposition 2.1, the Feynman–Kac formula. As a result, we can control the left tail of the perturbations. There exists an exponent $\zeta >0$, such that

2.42 $$\begin{aligned} \vert \mathcal {N}(p)(t,x) \vert&= \big \vert w(x) u(t,x) \cdot \big ( u(t,x) + v(t,x) \big ) \big \vert \nonumber \\&\le A(t,x) \cdot \big \vert p(t,x) \big \vert \nonumber \\&\le C e^{ \zeta x} \cdot \big \vert p(t,x) \big \vert &  \forall x \le B \le 0. \end{aligned}$$

In contrast, for $x \ge B$, we will use the general bound

2.43 $$\begin{aligned} \vert \mathcal {N}(p)(s,x) \vert&\le w(x) \cdot \vert p(t,x) \vert ^2 &  \forall x \in \mathbb {R}. \end{aligned}$$

Now choose any $\epsilon \le \delta _I$. We estimate (2.35), (2.36) and (2.37) separately, starting with

**1) The long-time case** $\varvec{t \ge 1}$

By Estimate (2.31) of Theorem 2.4, the linear Part (2.35) is bounded by

2.44 $$\begin{aligned} \Big \vert \int _{\mathbb {R}} G(t,x,y) p(0,y) dy \Big \vert \le C \frac{1+\vert x \vert }{(1+t)^{3/2}} \int _\mathbb {R} (1+\vert y\vert ) \cdot \vert p(0,y) \vert dy. \end{aligned}$$

For the moment, we only require that

2.45 $$\begin{aligned} P(0) := \vert \vert p(0, \cdot ) (1+ \vert \cdot \vert ) \vert \vert _{L^1(\mathbb {R})} \le 1, \end{aligned}$$

then the above expression is of order $(1+\vert x\vert )/(1+t)^{3/2}$.

Regarding the nonlinear part, we first consider the case $t-s \le 1$. For the back of the wave, we use the exponential Decay (2.42) and Estimate (2.30) of Theorem 2.4:

2.46 $$\begin{aligned}&\int _{t-1}^t ds \int _{-\infty }^{B} \big \vert \mathcal {G}(t-s,x,y) \big \vert \cdot \vert \mathcal {N} (p)(s,y) \vert dy \nonumber \\&\quad \le C \int _{t-1}^t ds \int _{-\infty }^{B} \big \vert \mathcal {G}(t-s,x,y) \big \vert \cdot e^{ \zeta y} \vert p(s,y) \vert \, dy \nonumber \\&\quad \le C \int _{t-1}^t ds \int _{-\infty }^{B} \big \vert \mathcal {G}(t-s,x,y)\big \vert \cdot e^{ \zeta y} \Theta (s) \frac{1+\vert y \vert }{(1+s)^{3/2}} \, dy\\&\quad \le C \Theta (t) \frac{1}{(1+t)^{3/2}} \int _{-\infty }^{B} \big \vert \mathcal {G}(t-s,x,y)\big \vert \cdot e^{ \zeta y} (1+\vert y \vert ) \, dy \nonumber \\&\quad \le C \Theta (t) \frac{1}{(1+t)^{3/2}} \cdot \sup _{y \le B} \big \{ e^{\zeta y} (1+\vert y \vert ) \big \}. \nonumber \end{aligned}$$

Note that by choosing $B \le 0$ sufficiently small, the supremum in the last term can be made arbitrarily small, which we need since the above expression is linear in $\Theta$.

For $x \ge B$, we use the fact $w(x) \cdot (1+\vert x \vert )^2$ is bounded in $\vert \vert . \vert \vert _\infty$. We apply Estimate (2.30) of Theorem 2.4:

2.47 $$\begin{aligned}&\int _{t-1}^t ds \int _{B}^\infty \big \vert \mathcal {G}(t-s,x,y)\big \vert \cdot \vert \mathcal {N} (p)(s,y) \vert \, dy \nonumber \\&\quad \le \int _{t-1}^t ds \int _{B}^\infty \big \vert \mathcal {G}(t-s,x,y) \big \vert \cdot \Theta (s)^2 \frac{(1+ \vert y \vert )^2}{(1+s)^{3}} w(y) \, dy \nonumber \\&\quad \le C \Theta (t)^2 \frac{1}{(1+t)^3} \int _{t-1}^t ds \int _{B}^\infty \big \vert \mathcal {G}(t-s,x,y)\big \vert \cdot (1+ \vert y \vert )^2 w(y) \, dy \\&\quad \le C_B \Theta (t)^2 \frac{1}{(1+t)^3}. \nonumber \end{aligned}$$

Now consider $t-s \ge 1$. Regarding $y \le B$, we again use Bound (2.42), and Estimate (2.31) of Theorem 2.4:

2.48 $$\begin{aligned}&\int _0^{t-1} ds \int _{-\infty }^{B} \big \vert \mathcal {G}(t-s,x,y)\big \vert \cdot \vert \mathcal {N} (p)(s,y) \vert \, dy \nonumber \\&\quad \le C \int _0^{t-1} ds \int _{-\infty }^B \big \vert \mathcal {G}(t-s,x,y) \big \vert \cdot e^{ \zeta y} \vert p(s,y) \vert \, dy \nonumber \\&\quad \le C \int _0^{t-1} ds \int _{-\infty }^B \big \vert \mathcal {G}(t-s,x,y) \big \vert \cdot e^{ \zeta y} \Theta (s) \frac{1+ \vert y \vert }{(1+s)^{3/2}} \, dy \\&\quad \le C \Theta (t) \cdot (1+ \vert x \vert ) \int _0^t \frac{1}{(1+t-s)^{3/2}} \frac{1}{(1+s)^{3/2}} \, ds \int _{-\infty }^B e^{\zeta y} (1+ \vert y \vert )^2 \, dy \nonumber \end{aligned}$$

We apply Lemma 2.5 to bound the integral over time:

2.49 $$\begin{aligned}&\le C \Theta (t) \frac{1+ \vert x \vert }{(1+t)^{3/2}} \int _{-\infty }^B e^{\zeta y} (1+ \vert y \vert )^2 \, dy. \end{aligned}$$

Again, note that we can make the last integral as small as we want if we shift *B* appropriately.

Regarding $y \ge B$, by Estimate (2.31) of Theorem 2.4:

2.50 $$\begin{aligned}&\int _0^{t-1} ds \int _{B}^{+\infty } \big \vert \mathcal {G}(t-s,x,y)\big \vert \cdot \vert \mathcal {N} (p)(s,y) \vert \, dy \nonumber \\&\quad \le \int _0^{t-1} ds \int _B^\infty \big \vert \mathcal {G}(t-s,x,y)\big \vert \cdot \Theta (s)^2 \frac{(1+ \vert y \vert )^2}{(1+s)^{3}} w(y) \, dy \nonumber \\&\quad \le C \Theta (t)^2 (1+ \vert x \vert ) \int _0^t \frac{1}{(1+t-s)^{3/2}} \frac{1}{(1+s)^3} \, ds \int _B^\infty (1+ \vert y \vert )^3 w(y) \, dy \nonumber \\&\quad \le C_B \Theta (t)^2 (1+ \vert x \vert ) \int _0^t \frac{1}{(1+t-s)^{3/2}} \frac{1}{(1+s)^3} \, ds \end{aligned}$$

We apply Lemma 2.5 to bound the integral over time, yielding

2.51 $$\begin{aligned}&\le C_B \Theta (t)^2 \frac{1+ \vert x \vert }{(1+t)^{3/2}}. \end{aligned}$$

Now, for all $t \ge 1$, combining our estimates (2.44), (2.47), (2.48), (2.49), (2.51) results in

2.52 $$\begin{aligned} \vert p(t,x) \vert&\le C P(0) \frac{1+ \vert x \vert }{(1+t)^{3/2}} \end{aligned}$$

2.53 $$\begin{aligned}&\quad + C \Theta (t) \frac{1}{(1+t)^{3/2}} \cdot \sup _{y \le B} \big \{ e^{\zeta y} (1+ \vert y \vert ) \big \} \end{aligned}$$

2.54 $$\begin{aligned}&\quad + C_B \Theta (t)^2 \frac{1}{(1+t)^{3/2}} \end{aligned}$$

2.55 $$\begin{aligned}&\quad + C \Theta (t) \frac{1+ \vert x \vert }{(1+t)^{3/2}} \int _{-\infty }^B e^{\zeta y} (1+ \vert y \vert )^2 \, dy \end{aligned}$$

2.56 $$\begin{aligned}&\quad + C_B \Theta (t)^2 \frac{1+ \vert x \vert }{(1+t)^{3/2}}. \end{aligned}$$

We now choose *B* sufficiently small such that the prefactors in (2.53) and (2.55), the terms that are linear in $\Theta (t)$, are both bounded by 1/8. Then, we can simplify the above to

2.57 $$\begin{aligned} \vert p(t,x) \vert&\le C P(0) \frac{1+ \vert x \vert }{(1+t)^{3/2}} + \frac{1}{4} \Theta (t) \frac{1+ \vert x \vert }{(1+t)^{3/2}} + C_B \Theta (t)^2 \frac{1+ \vert x \vert }{(1+t)^{3/2}}. \end{aligned}$$

Inserting our definition of $\Theta (t)$, see (2.34), we get

2.58 $$\begin{aligned} \Theta (t)&\le C P(0) + \frac{1}{4} \Theta (t) + C_B \Theta (t)^2 \qquad \forall t \ge 1. \end{aligned}$$

**2) The short-time case** $\varvec{t < 1}$

Estimate (2.30) of Theorem 2.4 yields the following bound for the linear Part (2.35):

2.59 $$\begin{aligned} \int _{\mathbb {R}} \big \vert \mathcal {G}(t,x,y)\big \vert \cdot \vert p(0,y) \vert \, dy \le C \cdot \, \vert \vert p(0,x) \vert \vert _\infty . \end{aligned}$$

For the nonlinear part, we again split the expression into two parts. This time, we use the point-wise Estimate (2.29) of Theorem 2.4, valid for short times:

2.60 $$\begin{aligned}&\int _0^{t-1} ds \int _{-\infty }^{B} \big \vert \mathcal {G}(t-s,x,y)\big \vert \cdot \vert \mathcal {N} (p)(s,y) \vert \, dy \nonumber \\&\quad \le \int _0^{t-1} ds \int _{-\infty }^{B} \big \vert \mathcal {G}(t-s,x,y) \big \vert \cdot e^{\zeta y} \vert p(s,y) \vert \, dy \nonumber \\&\quad \le \Theta (t) \frac{1}{(1+t)^{3/2}} \cdot \int _0^{t-1} ds \int _{-\infty }^{B} \big \vert \mathcal {G}(t-s,x,y) \big \vert \cdot e^{\zeta y} (1+ \vert y \vert ) \, dy\\&\quad \le C \Theta (t) \int _0^{t-1} ds \int _{-\infty }^{B} \frac{1}{t^{1/2}} e^{- \frac{ \vert x-y \vert ^2}{\kappa t} } e^{\zeta y} (1+ \vert y \vert ) \, dy. \nonumber \end{aligned}$$

The last integral is a short-time heat kernel applied to the exponentially decaying function $e^{\zeta x} (1+ \vert x \vert )$. We choose *B* appropriately such that the entire above expression is bounded by

2.61 $$\begin{aligned}&\le \frac{1}{16} \Theta (t). \end{aligned}$$

Considering $$y \ge B$$:

2.62 $$\begin{aligned}&\int _0^{t-1} ds \int _{B}^{+\infty } \big \vert \mathcal {G}(t-s,x,y)\big \vert \cdot \vert \mathcal {N} (p)(s,y)\vert \, dy \nonumber \\&\quad \le \int _0^{t-1} ds \int _{B}^{+\infty } \big \vert \mathcal {G}(t-s,x,y) \big \vert \cdot \Theta (s)^2 \frac{(1+ \vert y \vert )^2}{(1+s)^3} \, dy \nonumber \\&\quad \le C \Theta (t)^2 \int _0^{t-1} ds \int _{B}^{+\infty } \frac{1}{t^{1/2}} e^{- \frac{ \vert x-y \vert ^2}{\kappa t} } (1+ \vert y \vert )^2 \, dy \\&\quad \le C_B \Theta (t)^2. \nonumber \end{aligned}$$

In view of (2.59), (2.61), (2.62), we see that for all $t < 1$:

2.63 $$\begin{aligned} \vert p(t,x) \vert&\le C \, \vert \vert p(0,x) \vert \vert _{L^\infty (\mathbb {R})} + \frac{1}{16} \Theta (t) + C_B \Theta (t)^2. \end{aligned}$$

This implies

2.64 $$\begin{aligned} \Theta (t)&\le C \, \vert \vert p(0,x) \vert \vert _{L^\infty (\mathbb {R})} + \frac{1}{4} \Theta (t) + C_B \Theta (t)^2 \qquad \forall t <1. \end{aligned}$$

**3) Convergence given small initial data**

To control both the short-time Bound (2.64) and the large-time Bound (2.58), the initial data must be small in the sense that

2.65 $$\begin{aligned} Q(0) := \vert \vert p(x,0) \vert \vert _{L^\infty (\mathbb {R})} + \vert \vert p(x,0) \cdot (1+ \vert x \vert ) \vert \vert _{L^1(\mathbb {R})} \end{aligned}$$

is sufficiently small. We first choose a border $B \in \mathbb {R}$ such that both (2.64) and (2.58) are valid, resulting in

2.66 $$\begin{aligned} \Theta (t)&\le C Q(0) + \frac{1}{4} \Theta (t) + C_B \Theta (t)^2 \qquad \forall t \ge 0. \end{aligned}$$

Without loss of generality, we may assume that $C \ge 1$. Now choose any $0<\epsilon \le \delta _I$, with $\delta _I$ as in in (2.38), and consider initial data such that

2.67 $$\begin{aligned} 2 C Q(0)< \epsilon \quad \text {and} \quad 4 C C_B Q(0) < \frac{1}{2}. \end{aligned}$$

Then, at $$t = 0$$:

2.68 $$\begin{aligned} \Theta (0) = \sup _{x \in \mathbb {R}} \frac{ \vert p(x,0) \vert }{1+ \vert x \vert } \le Q(0)< 2C Q(0) < \epsilon . \end{aligned}$$

For such initial data, our critical Assumption (2.40) holds for small times $t>0$, since $\Theta (t)$ is continuous in *t*. Now suppose that there exists a first time $T\in (0, \infty )$ such that $\Theta (T) = 2CQ(0)$ for the first time. But then, by (2.66):

2.69 $$\begin{aligned} \Theta (t)&\le C Q(0) + \frac{1}{4} \Theta (t) + C_B \Theta (t)^2 \nonumber \\&\le C Q(0) + \frac{1}{4} 2 C Q(0) + C_B 4 C^2 Q(0)^2 \nonumber \\&\le C Q(0) + \frac{1}{2} C Q(0) + C Q(0) \big [ 4 C C_B Q(0) \big ]\\&< 2C Q(0), \nonumber \end{aligned}$$

a contradiction to our assumption. As a result, it holds that

2.70 $$\begin{aligned} \Theta (t)< 2CQ(0) < \epsilon \qquad \forall t \ge 0, \end{aligned}$$

which not only proves that the perturbation decays, but also shows that the necessary Bound (2.40) holds for all $t \ge 0$. $\square$

## Construction of the traveling waves

### Overview and notation

In the following, we refer to the Wave System (1.3) as $S_0$ if $d=0$, and to $S_d$ for $d >0$. We cite the result for the traveling waves of $S_0$:

#### Theorem 3.1

(Thm. 1.1 in Kreten (2022)) For $d = 0$ and $r \ge 0, c >0$, consider System $S_0$ (1.3). Set $i_c: = \max \{ 0, 1-c^2/4 \}$. For each pair $i_{-\infty }, i_{+\infty }\in \mathbb {R}^+_0$ such that

3.1 $$\begin{aligned} i_{+\infty }\in [i_c,1), \qquad i_{-\infty }= 2 - i_{+\infty }, \end{aligned}$$

there exists a unique non-negative traveling wave $a,i \in C^\infty (\mathbb {R}, \mathbb {R}^2)$ such that

3.2 $$\begin{aligned} \lim _{x \rightarrow \pm \infty }&a(x) = 0, \qquad \lim _{x \rightarrow \pm \infty } i(x) = i_{\pm \infty }. \end{aligned}$$

The function *i*(*x*) is decreasing, whereas *a*(*x*) has a unique local and global maximum. If $\frac{c^2}{4} + i_{+\infty }-1 = 0$, then the distance of the front to its limit behaves like $x \cdot e^{ - \frac{c}{2} x}$ asymptotically as $x \rightarrow + \infty$. If $\frac{c^2}{4} + i_{+\infty }-1 > 0$, then convergence as $x \rightarrow + \infty$ is purely exponential. Convergence as $x \rightarrow - \infty$ is purely exponential in all cases. The rates of convergence are

3.3 $$\begin{aligned} \begin{aligned} \mu _{- \infty }&= - \frac{c}{2} + \sqrt{ \frac{c^2}{4} + i_{\pm \infty } -1}>0 , \\ \mu _{+ \infty }&= \frac{c}{2} - \sqrt{ \frac{c^2}{4} + i_{\pm \infty } -1} >0. \end{aligned} \end{aligned}$$

Moreover, these are all bounded, non-negative, non-constant and twice differentiable solutions of Eq. (1.3) for $d = 0$.

**Fig. 3:**
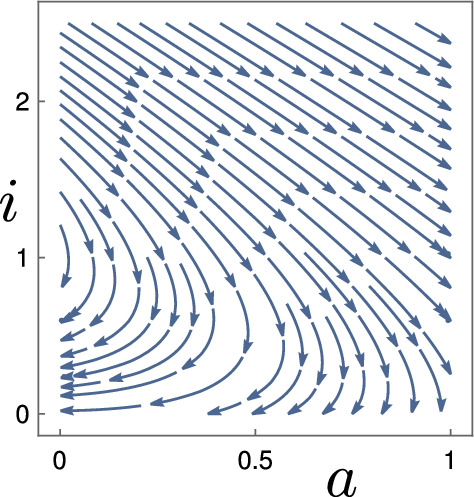
Two-dimensional representation of the family of traveling waves of $S_0$ for $c=2$ and $r=0$. A unique trajectory emerges from each point where $i_{-\infty }>1$, and converges to a limit where $i_{+\infty }<1$, where $i_{-\infty }+ i_{+\infty }= 2$. For $i_{-\infty }>2$, the trajectory eventually becomes negative (even though it still seems to converge)

The bound $i_{+\infty }\ge 1-c^2/4$ is classical for FKPP-fronts. If it is not fulfilled, the solutions spiral around their limit as $x \rightarrow + \infty$, see Sect. 3.2.1. Thus, they become negative and are physically irrelevant.

For the entire Sect. 3, keep the phase-plot of $S_0$ in Fig. 3 in mind. Qualitatively, we prove that this portrait remains valid for $S_d$: there exists a family of solutions, that continuously vary along the limits $i_{ \pm \infty }$. The only change is a perturbation estimate of type

3.4 $$\begin{aligned} i_{-\infty }+ i_{+\infty }= 2 + \mathcal {O}(d), \end{aligned}$$

which replaces the precise Statement (3.1) for $d=0$. Since we focus on the invading fronts, we consider only the case $c \ge 2$, the result is given in Theorem 3.18.

We transform the wave Eq. (1.3) into an equivalent System of ODEs. We denote differentiation w.r.t. *x* by a prime, and introduce two auxiliary variables $b=a'$ and $j=i'$. For $d \ne 0$, this system in coordinates $(a,b,i,j) \in \mathbb {R}^4$ reads

3.5 $$\begin{aligned} \frac{d}{dx} \begin{pmatrix} a \\ b \\ i \\ j \end{pmatrix} = \begin{pmatrix} b \\ a(a+i) -a -cb \\ j \\ - \frac{1}{d} [cj + ra + a(a+i)] \end{pmatrix}. \end{aligned}$$

By abuse of notation, we will denote $a'=b$ and $i'=j$, since introducing two auxiliary variables only obfuscates the system.

Section 3 is organized as follows: for $d >0$ and $K>1$, we analyze the unstable manifold of the fixed point $(a,a',i,i') = (0,0,K,0)$. It is one-dimensional and has one branch that is asymptotically non-negative, which we call $M^-_d(K)$. In Sect. 3.2, we will prove that any finite segment of $M^-_d(K)$ is continuous both in *d* and *K*. For $d>0$, this will follow from standard perturbation theory for dynamical systems. For passing from $d = 0$ to $d \sim 0$, we use geometric singular perturbation theory due to Jones (1995). In Sect. 3.3, we prove that a traveling wave $M^-_d(K)$ persists under small perturbations in *d*, up to its limit as $z \rightarrow + \infty$. The estimate $i_{-\infty }+ i_{+\infty }= 2 + \mathcal {O}(d)$ is proven in Sect. 3.4. We use all previous results to prove the existence of a family of non-negative traveling wave solutions in Sect. 3.5. Given the existence of these non-negative traveling waves, we conclude that there also must be an invading front among those.

### Dynamics around the fixed points

#### A degenerate linearization

At a fixed point $(a,a',i,i') = (0,0,K,0)$, the Jacobian of the system has Eigenvalues

3.6 $$\begin{aligned} \begin{aligned}&\lambda _1 = 0, &  \lambda _2 = -\frac{c}{d}, \\ &\lambda _3 = -\frac{c}{2} - \sqrt{ \frac{c^2}{4} + K -1}, &  \lambda _4 = - \frac{c}{2} + \sqrt{\frac{c^2}{4} + K -1 }. \end{aligned} \end{aligned}$$

The Eigenvalue $\lambda _2$ is new compared to the unperturbed system, all other Eigenvalues remain the same. The associated Eigenvectors are given by

3.7 $$\begin{aligned} \begin{aligned} e_1&= \begin{pmatrix} 0 \\ 0 \\ 1 \\ 0 \end{pmatrix}, \quad &  e_2 = \begin{pmatrix} 0 \\ 0 \\ -\frac{d}{c} \\ 1 \end{pmatrix}, \\ e_3&= \begin{pmatrix} - c\lambda _3 - d \cdot \lambda _3^2 \\ -c \cdot \lambda _3^2 - d \cdot \lambda _3^3 \\ K+r \\ \lambda _3 (K+r) \end{pmatrix}, &  e_4 = \begin{pmatrix} - c\lambda _4 - d \cdot \lambda _4^2 \\ -c \cdot \lambda _4^2 - d \cdot \lambda _4^3 \\ K+r \\ \lambda _4 (K+r) \end{pmatrix}. \end{aligned} \end{aligned}$$

For $K \in [0,1)$, corresponding to a possible limit as $x \rightarrow + \infty$, the Eigenvalues are real-valued if $K \ge 1-c^2/4$. For $K=0$, which is the limit of an invading front, we require that $c \ge 2$, otherwise converging trajectories can not stay non-negative: the *a*-component spirals around its limit 0 if $\lambda _3$ and $\lambda _4$ have an imaginary part.

For fixed $d >0$, we first analyze the behavior around a fixed point $(a,a',i,i') = (0,0,K,0)$ locally. The Jacobian of the system at the fixed point is degenerate due to the continuum of fixed points, we apply center manifold theory to work out the higher moments of the approximation. A practical introduction to this topic has been written by Carr (1982). If we ensure that all Eigenvectors (3.7) are distinct, they span the entire $\mathbb {R}^4$. In this case, the Jacobian can be diagonalized and the calculations are standard: no bifurcaction, neither in *d* nor *K*, occurs as long as all the Eigenvalues $\lambda _{2,3,4}$ remain real-valued and unequal zero.

If $K=1$, then $\lambda _4 = 0$, and if $K = 1 - c^2/4$, then $\lambda _3 = \lambda _4$ and their eigenspaces become colinear. If we exclude these two cases, the result is as intuitive as it is comfortable: the center manifold locally coincides with the set of fixed points, a defect linearization with three hyperbolic directions and one constant direction is the result, see Proposition 3.2. For the case $d = 0$, we present the rather standard calculations and the necessary changes of coordinates into the system of Eigenvectors in detail (Kreten 2022). The following result is completely analogue:

##### Proposition 3.2

For $d> 0,c>0$ and $K\in \mathbb {R}$ subject to the conditions

3.8 $$\begin{aligned} K \ne 1, \quad K \ne 1 - c^2/4, \end{aligned}$$

consider the fixed point (0, 0, *K*, 0) of $S_d$. Then, in a neighborhood of the fixed point, the center manifold of the fixed point coincides with the set of points

3.9 $$\begin{aligned} \{ a=a'=i'=0, \, i \in \mathbb {R} \}. \end{aligned}$$

Moreover, in this neighborhood, the flow $S_d$ is homeomorphic to

3.10 $$\begin{aligned} \begin{pmatrix} p' \\ u' \\ v ' \\ w' \end{pmatrix} = \begin{pmatrix} 0 \\ \lambda _2 \cdot u \\ \lambda _3 \cdot v \\ \lambda _4 \cdot w \end{pmatrix}, \end{aligned}$$

where *p*, *u*, *v*, *w* are the coordinates in the system of Eigenvectors (3.7).

Proposition 3.2 has two important implications. First, regarding the asymptotics as $x \rightarrow + \infty$, we can use Eq. (3.10) to deduce

##### Corollary 3.3

For $c>0, d >0$ and $K \in (1- \frac{c^2}{4},1)$, the fixed point (0, 0, *K*, 0) of $S_d$ is Lyapunov-stable, and trajectories asymptotically converge along $e_{2,3,4}$ (3.7).

##### Proof

Holds by (3.10), since $\lambda _2, \lambda _3, \lambda _4$ are real-valued and strictly negative. $\square$

#### Continuity with respect to the parameters

Regarding the asymptotics as $x \rightarrow - \infty$, we get

##### Corollary 3.4

For $d> 0, c>0, K>1$, the fixed point (0, 0, *K*, 0) of $S_d$ has a fast unstable manifold of dimension one, associated to the Eigenvalue

3.11 $$\begin{aligned} \lambda _4 = -\frac{c}{2} + \sqrt{ \frac{c^2}{4} + K -1 } > 0. \end{aligned}$$

This manifold has one branch such that $a,i >0$ asymptotically, which we denote as $M^-_d(K)$. Its asymptotic direction is given by

3.12 $$\begin{aligned} \frac{d}{dx} \begin{pmatrix} a \\ a' \\ i \\ i' \end{pmatrix} = \begin{pmatrix} c\lambda _4 + d \cdot \lambda _4^2 \\ c \cdot \lambda _4^2 + d \cdot \lambda _4^3 \\ -(K+r) \\ -\lambda _4 (K+r) \end{pmatrix}. \end{aligned}$$

Locally, the manifold $M^-_d(K)$ is continuous in *K*.

The local continuity of $M^-_d(K)$ w.r.t *K* can be extended to arbitrarily finite segments:

##### Corollary 3.5

Let $d> 0, K_0>1, c>0$ and choose any finite time-horizon $T \in \mathbb {R}$. Assume that the manifold $M^-_d(K_0,x)\vert _{x \in (- \infty , T]}$ exists, is smooth and bounded. Then, for *K* sufficiently close to $K_0$, each of the manifolds $M^-_d(K,x)\vert _{ x \in (- \infty , T]}$ is smooth and bounded, and they have a representation that is continuous in *K*.

##### Proof

The proof is a standard gluing argument: Fix some $K_0 >1$ and a finite time-horizon $T \in \mathbb {R}$. Assume that the manifold $M^-_d(K_0,x)\vert _{x \in (- \infty , T]}$ exists, is smooth and bounded. Consider a small ball $B_\delta$ of radius $\delta >0$ around the fixed point $(0,0,K_0,0)$, such that within $B_\delta$, the flow is equivalent to the linearized Flow (3.10). Define the exit time

3.13 $$\begin{aligned} x_\delta := \sup _{ x \in \mathbb {R} } \big \{ \forall s \le x : \, M^-_d(K_0,s) \in B_\delta \big \}, \qquad x^*:= \frac{x_\delta }{2}. \end{aligned}$$

As $K \rightarrow K_0$, the trajectories $M^-_d(K,x)\vert _{x \in (- \infty , x^*]}$ are continuous in *K*, due to the local statement 3.5. In particular, the points $M^-_d(K,x^*)$ converge to $M^-_d(K_0,x^*)$. We can now treat the rest of the trajectories

3.14 $$\begin{aligned} M^-_d(K,x)\vert _{x \in [x^*, T]} \end{aligned}$$

as initial value problems with converging initial data. Since $[x^*, T]$ is a finite time-interval, this follows from a Gronwall estimate for locally Lipschitz systems, check for example Theorem II-1-2 and Remark II-1-3 in the textbook of Hsieh and Sibuya (1999). $\square$

Similarly, continuity of $M^-_d(K_0,x)\vert _{x \in (- \infty , T]}$ w.r.t. *d* holds. The result for $d \gg 0$ is standard: local continuity follows from center manifold theory, see e.g. section 1.5 in the monograph of Carr (1982). Is is one of the fundamental tools for analyzing bifurcations, as explained by J. Guckenheimer and P. Holmes, see sections 3.2 and 3.4 in Guckheimer and Holmes (1983). The assumptions that the Eigenvectors (3.6) are distinct and that the Eigenvalues $\lambda _{2,3,4}$ are real-valued and non-zero are again crucial: they imply that locally in *K* and *d*, no bifurcation occurs. The local statement can easily be extended to arbitrary finite segments as before:

##### Proposition 3.6

Let $K>1, d_0>0, c >0$. For a finite time-horizeon $T \in \mathbb {R}$, assume that the manifold $M^-_{d_0}(K,x) \vert _{ x \in (-\infty ,T] }$ is smooth and bounded. There exists an open interval $I \subset \mathbb {R}^+_0, d_0 \in I$, such that for all $d \in I$, each of the manifolds $M^-_d(K,x)\vert _{ x \in (- \infty , T]}$ is smooth and bounded, and they have a representation that is continuous in *d*.

For passing from $d = 0$ to $d \sim 0$, we use geometric singular perturbation theory. The ODE-system with $d=0$ is three-dimensional, as it is independent of $i'$, but can be embedded into the $\mathbb {R}^4$, and then be perturbed smoothly when introducing a small diffusion $d > 0$. The resulting statement is analogue to Proposition 3.6, the proof is presented in Appendix D:

##### Corollary 3.7

Let $K>1, c >0, r \ge 0$. First consider the fixed point $(\bar{a},\bar{a}',\bar{i}) = (0,0,K)$ of $S_0$, together with its one-dimensional unstable manifold $M^-_0(K)$. Fix any semi-open interval $x \in (-\infty , T]$, where *T* is finite, and assume that $M^-_0(K,x)\vert _{x \in (-\infty , T]}$ is smooth and bounded. Lift it naturally into $\mathbb {R}^4$ via the fourth coordinate $i'=-a(a+i+r) /c$.

Now consider the perturbed system $S_d$. There exist some $d^*> 0$ such that for all $d \in (0,d^*)$: the fixed point $(a,a',i,i') = (0,0,K,0)$ has an adjacent one-dimensional unstable manifold $M^-_d(K,x)\vert _{x \in (-\infty , T]}$, that is continuous in *d* and converges to $M^-_0(K,x)\vert _{x \in (-\infty , T]}$ as $d \rightarrow 0$.

### Persistence of traveling waves under perturbation

We will not only prove the existence of non-negative traveling waves of $S_d$, but also that they all share certain monotonicity properties. We consider only the case $c \ge 2$, since this is regime in which an invading front can exist.

**Properties of traveling waves that are not invading fronts (TW)**: Let $K>1, d \ge 0, c >0$. Consider the manifold $M^-_{d}(K)$, and denote representing functions $a(x), a'(x), i(x), i'(x) \vert _{ x \in \mathbb {R}}$. We say that $M^-_{d}(K)$ admits the traveling wave properties **(TW)** if the following holds:

1. $$i'(x) < 0 \quad \forall x \in \mathbb {R}$$,
2. $$a(x) >0 \quad \forall x \in \mathbb {R}$$,
3. $$i(x) \ge i_{+\infty }>0 \quad \forall x \in \mathbb {R}$$,
4. The function *a*(*x*) has a unique local maximum, which is also the global one. At the phase-time of the maximum, it holds that $a+i \le 1$.
5. The trajectory converges monotonously to its limit as $x \rightarrow + \infty$. There exists a finite phase-time $x^*$, such that for all $x \ge x^*$:
  3.15 $$\begin{aligned} \begin{aligned} a'(x) < 0, \quad i''(x) > 0. \end{aligned} \end{aligned}$$

An invading front fulfills the same properties, with the exception that $\lim _{x \rightarrow + \infty }i(x) =0$. However, the assumption $i(x) \ge \gamma >0$ allows us to perturb the trajectory in such a way that the perturbed solutions stay non-negative. The properties **TW** have been proven for the non-negative traveling waves of $S_0$, as part of the proof of Theorem 3.1, see Kreten (2022). We prove that a given traveling wave persists under small perturbations in *d*:

#### Proposition 3.8

For $c \ge 2, d_0 \in [0, 3c/2)$ and $K \in (1,2]$, assume that the manifold $M^-_{d_0}(K)$ admits the wave-properties **TW**. Then, there exists an open interval $I \subset \mathbb {R}^+_0, d_0 \in I$, such that for all $d \in I$: the manifold $M^-_d(K)$ also admits **TW**.

The rest of Sect. 3.3 is devoted to the proof of Proposition 3.8. The required phase-space analysis is not relevant for the rest of the paper and can be skipped at first reading, we recommend to continue with Sect. 3.4.

#### Monotonicity, non-negativeness, and an attractor

Our analysis begins with the fact that *i*(*x*) must be monotone as long as the trajectory is non-negative:

##### Lemma 3.9

Let $K> 1, c>0$ and $d > 0$. Along the manifold $M^-_d(K,x)$, the inequality

3.16 $$\begin{aligned} i'(x) < 0 \end{aligned}$$

holds as long as $a(x) >0, i(x) \ge 0$.

##### Proof

By Corollary 3.5: $i'(x) < 0$ as $x \rightarrow - \infty$. We assume that there exists a finite phase-time $x^*$ such that $i'(x^*) = 0$ for the first time. This implies that $i(x^*) '' \ge 0$, since $i'(x)<0$ for all $x < x^*$. However, it also holds that $d i'' = -ci' - ra - a(a+i)$, which implies that $i''(x^*) <0$ if $i(x^*) \ge 0$ and $a(x^*) >0$. Thus, there can not be such a finite time $x^*$. $\square$

Since we do not change the equation for *a*(*x*) in Eq. (1.3), the following result - that traps *a*(*x*) within a non-negative region - can be taken over from the unperturbed system. This statement relies on an analysis of the subsystem for *a*(*x*) for fixed $i(x) = K$, and on the fact that *i*(*x*) is monotone. We consider only wave-speeds $c \ge 2$, to simplify the representation:

##### Proposition 3.10

(c.f. Thm. 6.1 and Prop. 6.4 in Kreten (2022)) For $c \ge 2, r \ge 0$ and $d \ge 0$, consider a solution of the Wave-Eq. (3.5), that at time $x=0$ is subject to the conditions

3.17 $$\begin{aligned} a(0)&>0,\end{aligned}$$

3.18 $$\begin{aligned} a'(0)&= 0, \end{aligned}$$

3.19 $$\begin{aligned} a(0) + i(0)&\le 1. \end{aligned}$$

Assume further that there exists some $T \in (0, +\infty ]$ such that

3.20 $$\begin{aligned} i (x) \ge 0, \, i'(x) \le 0 \quad \forall x \in [0, T). \end{aligned}$$

Then, *a*(*x*) is trapped in a non-negative attractor. It holds that

3.21 $$\begin{aligned} a(x) > 0 \text { and } a'(x) < 0 \qquad \forall x \in (0, T). \end{aligned}$$

Moreover, if for some $\kappa \in (0,1)$ and $x_\kappa \in (0, T)$, it holds that

3.22 $$\begin{aligned} a(x_\kappa ) + i (x_\kappa ) = 1-\kappa , \end{aligned}$$

then there exists a finite constant $L_\kappa \ge 0$, that depends only on $\kappa$, such that

3.23 $$\begin{aligned} \int _{s_1}^{s_2} a(x) \, dx&\le L_\kappa \cdot a(s_1) \quad \forall \, 0 \le s_1 \le s_2 \le T. \end{aligned}$$

Lemma 3.9 and Proposition 3.10 imply that along $M^-_d(K,x)$, we only have prove that *a*(*x*) reaches a local maximum, and that $i(x) \ge 0$ for all $x \in \mathbb {R}$, then convergence and non-negativeness follow. As $i'(x)$ essentially depends on *a*(*x*), Inequality (3.23) will be handy for proving that $i'(x) \rightarrow 0$. For $S_0$, we did this in chapters 6.3 and 6.4 of Kreten (2022). For $S_d$, the new term $di''$ needs to be dealt with, see the following Sect. 3.3.2. Before, we conclude this section with another simple phase space argument:

##### Lemma 3.11

Let $K> 1, c>0, r \ge 0$ and $d > 0$. Consider the manifold $M^-_d(K,x)$. If there exists some $T \in \mathbb {R}$ such that

3.24 $$\begin{aligned} i(x)&\ge 0 \quad \forall x \in (-\infty ,T], \end{aligned}$$

then *a*(*x*) has at most one local maximum, say at some phase-time $x_0 \in (-\infty ,T]$. There, it holds that $a(x_0)+i(x_0) \le 1$. Moreover:

3.25 $$\begin{aligned} a(x)&> 0, \, i'(x) < 0 \quad \forall x \in (-\infty ,T]. \end{aligned}$$

If $T = +\infty$, then both *a*(*x*) and *i*(*x*) converge and stay non-negative.

##### Proof

The manifold $M^-_d(K)$ leaves the fixed point in positive direction of *a* and negative direction of *i*. We have already proven that $i'(s)<0$ as long as $a>0, i \ge 0$. Assume that *a*(*s*) has a first local maximum at some $x_0 \in \mathbb {R}$. There, it holds that

3.26 $$\begin{aligned} 0 \ge a''(x_0) = a(x_0) \cdot \big (a(x_0)+i(x_0) - 1 \big ), \end{aligned}$$

which implies that $$a(x_0)+i(x_0)\le 1$$, since $$a(x_0) >0$$. But since $$i(s) \ge 0$$ for all $$s \in (-\infty ,x]$$, we can apply Proposition 3.10 to trap $$a(s)\vert _{x \in (x_0,x]}$$: along this part of the trajectory, it holds that $$a(x) >0$$ and $$a'(x) <0 $$. $$\square $$

#### The tail of a perturbed trajectory

For the entire Sect. 3.3.2, we work under the assumption that a reference trajectory $M^-_{d_0}(K)$ exists for some $d_0 \ge 0$, which we perturb when changing $d \sim d_0$:

**Assumption**: Let $K>1, c \ge 2, r \ge 0$ and $d_0 \ge 0$. Assume that the manifold $M^-_{d_0}(K)$ admits properties **TW**, and choose four representing functions

3.27 $$\begin{aligned} \bar{a}(x), \bar{a}'(x), \bar{i}(x), \bar{i}'(x), \quad x \in \mathbb {R}. \end{aligned}$$

Given $M^-_{d_0}(K)$, we vary the parameter *d* and track the perturbed trajectories. Therefore, we denote the representing functions of the manifolds $M^-_{d}(K)$ as

3.28 $$\begin{aligned} a_d(x), a'_d(x), i_d(x), i'_d(x), \quad x \in \mathbb {R}, \end{aligned}$$

to emphasize their dependency on *d*.

The results of Proposition 3.6 and Corollary 3.7 are structurally similar: they yield continuity of arbitrarily large, but finite time-horizons of $M^-_d(K)$, when varying $d \ge 0$, resulting in the following Proposition 3.12. It remains to control the tail as $x \rightarrow + \infty$ (for the result, see Proposition 3.8: the wave proverties **TW** persist for *d* close to $d_0$).

##### Proposition 3.12

Let the manifold $M^-_{d_0}(K)$ be as above and choose a finite time-horizon $T \in \mathbb {R}$. For all $\epsilon >0$, there exists an open interval $I \subset \mathbb {R}^+_0, d_0 \in I$, such that for all $d \in I$: the manifold $M^-_d(K,x)\vert _{x \in (-\infty ,T]}$ is of distance at most $\epsilon$ to $M^-_{d_0}(K,x)\vert _{x \in (-\infty ,T]}$ and is strictly non-negative.

##### Proof

For the reference trajectory, note that there exists a $\gamma >0$ such that

3.29 $$\begin{aligned} \bar{i}(x) \ge \gamma \quad \forall x \in (-\infty ,T]. \end{aligned}$$

If $d_0 = 0$, apply Corollary 3.7, if $d_0>0$ apply Proposition 3.6, both yield continuity in *d*: for all $\epsilon >0$, there exists an open interval $I \subset \mathbb {R}^+_0, d_0 \in I$ such that for all $d \in I$:

3.30 $$\begin{aligned} \big \vert \big \vert M^-_d(K,x) - M^-_{d_0}(K,x) \big \vert \big \vert _\infty&\le \epsilon \quad &  \forall x \in (-\infty ,T]. \end{aligned}$$

In particular, for $\epsilon \le \gamma /2$:

3.31 $$\begin{aligned} i_d(x) \ge \bar{i}(x) - \epsilon&\ge \gamma - \gamma /2 > 0 \quad &  \forall x \in (-\infty ,T]. \end{aligned}$$

By Lemma 3.11, this implies also positiveness of $a_d(x)\vert _{x \in (-\infty ,T]}$. $\square$

We trap $M^-_d(K)$ in the attractor from Proposition 3.10:

##### Lemma 3.13

Let $M^-_{d_0}(K)$ as before. There exists a constant $\kappa \in (0,1)$, an open interval $I_0 \subset \mathbb {R}^+_0, d_0 \in I_0$, and a finite phase-time $x_\kappa$, such that for all $d \in I_0$:

1. the unstable manifold $M^-_d(K,x)\vert _{x \in (-\infty ,x_\kappa ]}$ is strictly positive and has a unique first local maximum of active particles at a finite phase-time $\tilde{x}_0(d) \in (-\infty , x_\kappa ]$.
2. It holds that
  $$\begin{aligned} a_d'(x_\kappa ) < 0, \quad a_d(x_\kappa ) + i_d(x_\kappa )&\le 1 - \kappa . \end{aligned}$$

##### Proof

On $M^-_{d_0}(K,x)$, there exists a unique sharp global maximum of $\bar{a}(x)$, say at some phase-time $x_0$. Choose a phase-time $x_\kappa > \tilde{x}$ so large, such that

3.32 $$\begin{aligned} \bar{a}(x_\kappa ) + \bar{i}(x_\kappa )&\le 1 - 2 \kappa , \quad \hbox { for some}\ \kappa \in (0,1), \end{aligned}$$

3.33 $$\begin{aligned} \bar{a}'(x_\kappa )&\le 2 \delta < 0, \quad \text { for some }\delta >0, \end{aligned}$$

which must exist since $M^-_{d_0}(K,x)$ admits **TW**.

Apply the previous Proposition 3.12 over the interval $(-\infty , x_\kappa ]$, such that we can control $M^-_d(K,x)\vert _{x \in (-\infty , x_\kappa ]}$ for *d* in some open interval $I \subset \mathbb {R}^+_0$. We choose $\vert d-d_0 \vert$ sufficiently small such that

3.34 $$\begin{aligned} i_d(x)&> 0 \quad \forall x \in (- \infty , x_\kappa ], \end{aligned}$$

3.35 $$\begin{aligned} a_d(x_\kappa ) + i_d(x_\kappa )&\le 1 - \kappa , \end{aligned}$$

3.36 $$\begin{aligned} a_d'(x_\kappa )&\le \delta < 0. \end{aligned}$$

By Corollary 3.5: $a_d'(x) >0$ as $x \rightarrow - \infty$. Hence, also $a_d(x)$ must have a local maximum before $x_\kappa$. It is unique since $i_d(x)\vert _{x \in (-\infty ,x_\kappa ]} > 0$ and by Lemma 3.11. $\square$

In view of Proposition 3.10, we now have trapped $M^-_d$ in a non-negative monotone attractor, as long as we can control $i_d \ge 0$. We first prove monotonicity of the tail as $x \rightarrow + \infty$:

##### Lemma 3.14

Consider $M^-_{d_0}(K), I_0$ and $x_0$ as in Lemma 3.13. There exists a phase-time $x_{tail} \ge x_\kappa$, such that for all $x^*\in ( x_{tail}, \infty )$:

There exists an open interval $I_1 \subset I_0, d_0 \in I_1$, such that for all $d \in I_1$:

3.37 $$\begin{aligned} a_d(x^*),i_d(x^*)&>0, \nonumber \\ a_d'(x^*), i_d'(x^*)&< 0, \\ i_d''(x^*)&>0. \nonumber \end{aligned}$$

Moreover, given the existence of such an $x^*$, it holds for all $x \ge x^*$, and as long as $i_d(x) \ge 0$:

3.38 $$\begin{aligned} i_d''(x)&> 0, \end{aligned}$$

and thus all $a_d, a'_d,i_d,i'_d$ converge monotonously to zero.

##### Proof

The Inequalities (3.37) are true on the tail of $M^-_{d_0}$, say for all $x \ge x_1$, where we let $x_1 \ge x_\kappa$ without loss of generality. Now pick some $x_2 > x_1$. There exists an open interval $I_1 \subset I_0, d_0 \in I_1$, such that for all $d \in I_1$:

The manifold $M^-_d(K,x)\vert _{x \in (-\infty ,x_2]}$ exists, is non-negative and converges to $M^-_0(K,x)\vert _{x \in (-\infty ,x_2]}$ as $d \rightarrow d_0$. By continuity: for $\vert d -d_0 \vert$ sufficiently small and for all $x \in [x_1, x_2]$:

3.39 $$\begin{aligned} a_d(x),i_d(x) >0, a'_d(x)<0, i'_d(x) <0. \end{aligned}$$

On $[x_1, x_2]$: $\bar{i}''(x) > 0$, so $\bar{i}'(x_1) < \bar{i}'(x_2)$. Since $i'_d \rightarrow \bar{i}'$, there must also be some $x^*_d \in [x_1, x_2]$, such that $i_d''(x^*_d) >0$, for all *d* such that $\vert d - d_0 \vert$ is sufficiently small.

We assume that there exists a finite time $x_3 \ge x^*_d$ such that $i''_d(x_3) = 0$ for the first time after $x^*$. This implies that $i'''_d(x_3) \le 0$. On the other hand, deriving the equation for *i* in (1.3) once yields

3.40 $$\begin{aligned} \begin{aligned} d \cdot i'''&= -(ci' + ra + a(a+i))' \\&= -\big (ci'' + ra' + a'(a+i) + a(a'+i') \big ) \\&= -ra' - a'(a+i) - a(a'+i'). \end{aligned} \end{aligned}$$

As long as $i_d \ge 0$, we can apply Lemmas 3.11 and 3.9. Then, all three terms in the last line are strictly positive, resulting in $i_d'''(x_3) > 0$, contradicting our assumption. This allows to fix $x_{tail} = x_2$, independent of *d*. $\square$

*Remark*: Note that we can choose $x^*$ from Lemma 3.14 as large as we want, the price is a stronger restriction regarding $d \sim d_0$. This will be helpful if we assume that $M^-_{d_0}(K)$ converges: also the perturbed trajectories get as close to the limit as we need.

Given monotonicity of the tail, we can control $i_d(x)$ for $x \in [x^*, \infty )$:

##### Corollary 3.15

Consider $M^-_{d_0}(K)$ and $I_1,x^*$ as in Lemma 3.14. There exists a finite constant $J \ge 0$, such that for all $d \in I_1$ and all phase-times $x_2 \ge x_1 \ge x^*$, the bound

3.41 $$\begin{aligned} \begin{aligned} c \cdot \big \vert \big ( i_d(x_1) - i_d(x_2) \big ) \big \vert \le d \cdot \vert i_d'(x_1) \vert + J \cdot a_d(x_1) \end{aligned} \end{aligned}$$

holds as long as $i_d(x) \ge 0$ for all $x \in ( - \infty , x_2]$.

##### Proof

Choose $x^*$ as in the previous Lemma 3.14. For $x_2 \ge x_1 \ge x^*$, integrate $0 = d i_d'' + c i_d' + a_d(a_d+i_d+r)$:

3.42 $$\begin{aligned} c \cdot \big \vert i_d(x_2) - i_d(x_1) \big \vert&= \big \vert d \cdot \big (i_d'(x_2) - i_d'(x_1) \big ) + \int _{x_1}^{x_2} a_d(a_d+i_d+r) \, ds \, \big \vert . \end{aligned}$$

As long as $i_d \ge 0$, Proposition 3.10 and the previous Lemmas 3.11, 3.9 and 3.14 imply $a_d \ge 0, a_d'\le 0, i_d' \le 0$ and $i_d'' \le 0$. Thus, since $a_d+i_d+r \le 1+r$:

3.43 $$\begin{aligned} c \cdot \big \vert i_d(x_2) - i_d(x_1) \big \vert&\le d \cdot \big \vert i_d'(x_1) \big \vert + (1+r) \cdot \int _{x_1}^{x_2} a_d(x) \, dx. \end{aligned}$$

By our bound $a_d(x_\kappa ) + i_d(x_\kappa ) \le 1 - \kappa$, for some $x_\kappa \le x^*$, Proposition 3.10 implies that there exists a constant $L_\kappa \ge 0$ such that

3.44 $$\begin{aligned} \int _{x_1}^{x_2} a(s) \, ds&\le L_\kappa \cdot a_d(x_1) \qquad \forall x_2 \ge x_1 \ge x_0, \end{aligned}$$

as long as $i_d(x) \ge 0$ for all $x \in (-\infty , x_2]$. $\square$

We now prove the existence of a non-negative traveling wave for $d \sim d_0$, all that is left to be done is to prove convergence as $x \rightarrow + \infty$:

##### Proof of Proposition 3.8

Consider a manifold $M^-_{d_0}(K)$ that fulfills **TW**. Then, there exists a constant $\gamma >0$, such that $\bar{i}(x) \ge \gamma$ for all $x \in \mathbb {R}$. We prove that for all *d* sufficiently close to $d_0$:

3.45 $$\begin{aligned} \vert i_d (x) - \bar{i}(x) \vert \le \frac{\gamma }{2} &  \forall x \in \mathbb {R}. \end{aligned}$$

This implies that $i_d(x) \ge \frac{\gamma }{2} >0$, and Lemma 3.9 yields positiveness and convergence of $M^-_d(K)$. The rest of the wave properties **TW** then follows by Lemma 3.9.

Let $x^*$ as in Corollary 3.15: there exists an open interval $I_1 \subset \mathbb {R}_0^+$ such that for all $d \in I_1$: the trajectory $M^-_d(K,x) \vert _{x \in (-\infty ,x^*]}$ is non-negative, and there exists some $J \ge 0$ such that for all $x_2 \ge x_1 \ge x^*$:

3.46 $$\begin{aligned} i_d(x_2) \ge i_d(x_1) - \frac{d}{c} \vert i'_d(x_1) \vert - \frac{J}{c} \cdot a_d(x_1), \end{aligned}$$

as long as $i_d \ge 0$. Now, choose $x_1 \ge x^*$ large enough such that both

3.47 $$\begin{aligned}&\frac{J}{c} \cdot \bar{a}(x_1) \le \delta : = \frac{\gamma }{7}, \end{aligned}$$

3.48 $$\begin{aligned}&\vert \bar{a}(x_1)\vert + \vert \bar{a}'(x_1)\vert + \vert \bar{i}'(x_1)\vert + \vert \bar{i}(x_1) - \gamma \vert \le \delta : = \frac{\gamma }{7}, \end{aligned}$$

which is possible since $M^-_{d_0}(K)$ converges. Then choose an even smaller open interval $I_2 \subset I_1, d_0 \in I_2$, such that by continuity: for all $d \in I_2$, $M^-_d(K,x)\vert _{x \in (- \infty , x_1]}$ is non-negative and of distance at most

3.49 $$\begin{aligned} \epsilon = \frac{\gamma }{7(1+\frac{J}{C})} \end{aligned}$$

to $M^-_{d_0}(K,x)\vert _{x \in (- \infty , x_1]}$. Then, in view of Eqs. (3.47) and (3.48), also

3.50 $$\begin{aligned}&\frac{J}{c} \cdot a_d(x_1) \le \frac{2 \gamma }{7} = 2 \delta , \end{aligned}$$

3.51 $$\begin{aligned}&\vert a_d(x_1) \vert + \vert a'_d(x_1) \vert + \vert i'_d(x_1) \vert \le \frac{ 2 \gamma }{7} = 2 \delta . \end{aligned}$$

This implies that for all $d \in I_2$ and $x_2 \ge x_1$, and by Eq. (3.46):

3.52 $$\begin{aligned} \begin{aligned} i_d(x_2)&\ge i_d(x_1) - \frac{d}{c} \Big \vert i'_d(x_1) \Big \vert - \frac{J}{c} \cdot a_d(x_1) \\&\ge \bar{i}(x_1) - \epsilon - \frac{d}{c} \Big \vert \bar{i}'(x_1) + \epsilon \Big \vert - 2 \delta \\&\ge \gamma - \frac{\gamma }{7} - \frac{d}{c} \cdot \Big \vert \frac{\gamma }{7} + \frac{\gamma }{7} \Big \vert - 2 \frac{\gamma }{7} \\&\ge \frac{ 4\gamma }{7} - \frac{ d }{c} \cdot \frac{2\gamma }{7}. \end{aligned} \end{aligned}$$

The last line is strictly positive for $d < 3c/2$. Thus, $i_d(x_2) \ge 0$ for all $x_2 \ge x_1$, and the trajectory stays non-negative and converges monotonously to its limit, as claimed. $\square$

### The mapping of the limits

In view of the previous Section, the wave properties **TW** persist under small perturbations in *d* as long as $i_{+\infty }> 0$. However, we can relate the limits of any bounded and non-negative solution up to a term of order $\mathcal {O}(d)$:

#### Proposition 3.16

For $d > 0$, the two limits $i_{-\infty }$ and $i_{+\infty }$ of any smooth, bounded and non-negative traveling wave (where $a \not \equiv 0$) fulfill

3.53 $$\begin{aligned} 2 - d \cdot \frac{2(r+1)}{c^2}< i_{-\infty }+ i_{+\infty }< 2 . \end{aligned}$$

For the proof, we first verify integrability of any non-negative traveling wave:

#### Lemma 3.17

For $d > 0$, let $a(x),a'(x),i(x),i'(x)\vert _{ x \in \mathbb {R}}$ be a smooth, bounded and non-negative solution of System (3.5), where $a \not \equiv 0$. Then, as $x \rightarrow \pm \infty$, *a*(*x*) vanishes and *i*(*x*) converges, and $a,a',a'',i',i'' \in L^1(\mathbb {R})$. Moreover, *i*(*x*) is not constant and $i' \le 0$. The function *a*(*x*) has a unique global and local maximum, and $a(x) >0$ for all $x \in \mathbb {R}$.

#### Proof

Let $a(x),b(x),i(x),i'(x)$ as above. If *i*(*x*) does not converge at either $x \rightarrow + \infty$ or $x \rightarrow - \infty$, it must oscillate: in this setting, we can use the reasoning in the proof of Lemma 3.9 to provoke a contradiction. Thus, *i*(*x*) is either increasing or decreasing. If $i'(x) \ge 0$ for all $x \in \mathbb {R}$, then

3.54 $$\begin{aligned} - d \cdot i'' \ge i' + a(a+i+r) \ge 0. \end{aligned}$$

As a consequence, the trajectory can not be bounded, since $i'(x)$ can not vanish at both $x \rightarrow \pm \infty$, or *i*(*x*) must be constant. However, if *i*(*x*) was constant, then

3.55 $$\begin{aligned} 0 \equiv d i'' + ci' = -a(a+i+r), \end{aligned}$$

which can not hold since we assumed that $a \not \equiv 0$. It follows that *i*(*x*) is decreasing and by boundedness must converge as $x\rightarrow \pm \infty$, so $i' \in L^1(\mathbb {R})$. The two limits must be fixed points, we denote them as $(0,0,i_{-\infty },0)$ and $(0,0,i_{+\infty },0)$.

Assume that at some finite phase-time $x^*$: $a(x^*) = 0$. Since we assumed that $a \ge 0$, this must be a local minimum, so $a'(x^*) = 0$. Then also $-a''(x^*) = ca'(x^*) + a(x^*) -a(x^*)(a(x^*)+i(x^*)) = 0$, and by induction: $a^{(n)}(x^*) = 0$ for all $n \in \mathbb {N}$. But this contradicts the assumption $a \not \equiv 0$.

For $a(x) \not \equiv 0$, there is at least one local maximum of active particles, since the trajectory converges at both ends. We denote this maximum as $(a_0,0,i_0,i'_0)$. At this point, $a'' = a_0 (a_0 + i_0 -1) \le 0$, so $a_0 + i_0 \le 1$, since $a_0 >0$. Assume that there is also a local minimum of $a(x)$, denoted as $(a_m,0,i_m,i_m')$. Since $a(x)$ vanishes at both ends, we may assume without loss of generality that this be the first local minimum after passing through $(a_0,0,i_0,i_0')$.

Now, since $a_m(a_m + i_m-1-1) =a_m'' \ge 0$, it must hold that $a_m + i_m \ge 1$. But $i(x)$ is decreasing, so $a(x)$ must have been increasing, a contradiction to the assumption that this is the first local minimum after the maximum $(a_0,0,i_0,i_0')$. As a consequence, there is only one local maximum of active particles, which is also the global one. Further, this implies $a' \in L^1(\mathbb {R})$.

Given that $i' \in L^1(\mathbb {R})$, we know that the expression

3.56 $$\begin{aligned} \int _{\mathbb {R}} d \cdot i(x)'' + a(x)\big [a(x)+i(x)+r\big ] dx = c ( i_{-\infty }- i_{+\infty }) \end{aligned}$$

is finite, however the left side might only exist in a Riemannian sense. Integrating the left-hand side over a finite interval $[-M,M]$, it also holds that

3.57 $$\begin{aligned} \begin{aligned}&\int _{-M}^{M} d \cdot i(x)'' + a(x)\big [a(x)+i(x)+r\big ] dx\\&\quad = d \big [ i'(M) - i'(-M) \big ] + \int _{-M}^{M} a(x) \big [a(x)+i(x)+r\big ] dx. \end{aligned} \end{aligned}$$

By monotonicity of *i*(*x*), it holds that $i'(x) \rightarrow 0$ as $x \rightarrow \pm \infty$. By (3.56) and (3.57):

3.58 $$\begin{aligned} \begin{aligned} c ( i_{-\infty }- i_{+\infty }) = \int _{\mathbb {R}} a(x) \big [a(x)+i(x)+r \big ] dx, \end{aligned} \end{aligned}$$

and the right-hand side is integrable, since all terms are non-negative. In view of (3.57), also $i''(x)$ is integrable, as a sum of integrable terms. We proceed in a similar fashion with the equation $a''+ca' +a = a(a+i)$. We integrate it over the finite interval $[-M,M]$:

3.59 $$\begin{aligned} \int _{-M}^{M} a''(x) + c a'(x) + a(x) \, d x= \int _{-M}^{M} a(x) \cdot \big [ a(x) + i(x) \big ] d x. \end{aligned}$$

We already know that the right-hand is integrable, and that both $a(\pm M)$ and $a'(\pm M)$ vanish as $M \rightarrow +\infty$. This implies

3.60 $$\begin{aligned} \begin{aligned} \int _{\mathbb {R}} a(x) dx&= \lim _{M \rightarrow + \infty } \Big [ a'(M) -a'(-M) + c \big [a(M) - a(-M) \big ] + \int _{-M}^{M} a(x) d x\Big ] \\&= \int _{\mathbb {R}} a(x) \cdot \big [ a(x)+i(x) \big ] \, dx. \end{aligned} \end{aligned}$$

Hence also *a*(*x*) is integrable, since it is non-negative. Finally, as a sum of integrable terms, also $a''(x)$ is integrable. $\square$

#### Proof of Proposition 3.16

Given integrability a non-negative traveling wave, we define

3.61 $$\begin{aligned} A (x) := \int _{- \infty }^x a(s), \quad \mathcal {A}:= A( + \infty ). \end{aligned}$$

Then, integrating the equation for *a*(*x*) in (1.3), it must hold that

3.62 $$\begin{aligned} \begin{aligned} \mathcal {A}&= \int _\mathbb {R} a(x)\big [a(x)+i(x)\big ] dx >0, \\ c \cdot \big ( i_{-\infty }- i_{+\infty }\big )&= r \cdot \mathcal {A} + \int _{ \mathbb {R} } a(x)\big [a(x)+i(x)\big ] dx. \end{aligned} \end{aligned}$$

Moreover, by integration by parts:

3.63 $$\begin{aligned} \begin{aligned}&\int _{\mathbb {R}} a(x)\big [a(x)+i(x)\big ] dx \\&\quad = \mathcal {A} \cdot i_{+\infty }- \int _{\mathbb {R}} A(x) \big [ a'(x)+i'(x) \big ]dx \\&\quad = \mathcal {A} \cdot i_{+\infty }+ \frac{1}{c} \int _{\mathbb {R}} A(x) \big [ (1+r) a(x) + d i''(x) + a''(x) \big ] dx\\&\quad = \mathcal {A} \cdot i_{+\infty }+ \frac{1+r}{2c} \mathcal {A}^2 + \frac{1}{c} \int _{\mathbb {R}} A(x) \cdot a''(x) \, dx + \frac{d}{c} \int _{\mathbb {R}} A(x) \cdot i''(x) \, dx\\&\quad = \mathcal {A} \cdot i_{+\infty }+ \frac{1+r}{2c} \mathcal {A}^2 - \frac{ d}{c} \int _{\mathbb {R}} a(x) \cdot i'(x) \, dx. \end{aligned} \end{aligned}$$

Using this and Eq. (3.62), it follows that

3.64 $$\begin{aligned} \mathcal {A}&= \frac{c}{1+r} \cdot ( i_{-\infty }- i_{+\infty }), \end{aligned}$$

3.65 $$\begin{aligned} \mathcal {A}&= \mathcal {A} \cdot i_{+\infty }+ \frac{1+r}{2c} \mathcal {A}^2 - \frac{d}{c} \cdot \int _{\mathbb {R}} a(x) \cdot i'(x) \, dx. \end{aligned}$$

We know that $\int _{\mathbb {R}} a \cdot i' < 0$. We drop this term in Eq. (3.65), divide by $\mathcal {A} >0$ and then eliminate the variable $\mathcal {A}$ via (3.64), leading to

3.66 $$\begin{aligned} 1 > i_{+\infty }+ \frac{1}{2}(i_{-\infty }- i_{+\infty }). \end{aligned}$$

The first part of the claim follows: $2 > i_{+\infty }+ i_{-\infty }$.

At the local maximum of *a*(*x*), it holds that $a < a + i \le 1$, see the proof of Lemma 3.11. Thus $-i' \ge -ai' \ge 0$, and we can bound (using (3.64)):

3.67 $$\begin{aligned} -\frac{d}{c} \int _{\mathbb {R}} a(x) \cdot i'(x) \, dx < \frac{d}{c} \cdot ( i_{-\infty }- i_{+\infty }) = \frac{d}{c} \cdot \frac{1+r}{c} \mathcal {A}. \end{aligned}$$

As before, we apply this bound to (3.65), divide by $\mathcal {A}$ and eliminate $\mathcal {A}$ via (3.64), which leads to

3.68 $$\begin{aligned} 2 < i_{-\infty }+ i_{+\infty }+ 2\frac{d (1+r)}{c^2}. \end{aligned}$$

$$\square $$

*Remark*: These estimates seem to be sharp only asymptotically for $d \sim 0$, see the numerical Table B1 in Appendix B.

### Existence of the traveling waves

The existence of an invading front is proven in two steps: we first prove that for $d >0$, there exists a traveling wave that is non-negative, but not necessarily a traveling front, as long as we can control the limits via the $\mathcal {O}(d)$-Estimate (3.53). Afterwards, we show that for fixed $d \ge 0$, the existence of an arbitrary non-negative traveling wave also implies the existence of an invading front, by using the continuum of possible limits.

#### Arbitrary bounded non-negative solutions

##### Theorem 3.18

(Existence of a traveling wave solution) Let $r \ge 0, c \ge 2$ and $i_{-\infty }\in (1,2)$. Then, for all $d > 0$ that fulfill

3.69 $$\begin{aligned}&d < \frac{3c}{2}, \end{aligned}$$

3.70 $$\begin{aligned}&d \frac{2(r+1)}{c^2} \le 2 - i_{-\infty }, \end{aligned}$$

there exists a non-negative traveling wave with limits

3.71 $$\begin{aligned} \lim _{x \rightarrow \pm \infty } a(x)&= 0, \end{aligned}$$

3.72 $$\begin{aligned} \lim _{x \rightarrow - \infty } i(x)&= i_{-\infty }, \end{aligned}$$

3.73 $$\begin{aligned} \lim _{x \rightarrow + \infty } i(x)&> 2 - i_{-\infty }- d \frac{2(r+1)}{c^2} \ge 0. \end{aligned}$$

All these waves admit the wave properties **TW**. Moreover, for $d \in (0,1)$, the waves converge exponentially fast. The rates of convergence depend on $i_{\pm \infty }$ and are given by (3.3), as in the case $d=0$.

##### Proof

Fix $i_{-\infty }\in (1,2)$, and consider the manifold $M^-_d(i_{-\infty })$ as before. We prove **TW** for all *d* as claimed. For $d = 0$, this statement is part of the proof of Theorem 3.1. We will use continuity in *d*, see Proposition 3.8, for which we need Bound (3.69). The second Bound (3.70) is the one from Proposition 3.16, it will ensure that $\lim _{x \rightarrow + \infty } i(x) > 0$. The rates of convergence will be discussed when finishing the proof of Theorem 1.1, at the end of Sect. 3.5.2.

Since **TW** is true for $d = 0$, it is also true for $d \in [0, d_1)$, for some $d_1 >0$, by Proposition 3.8. Let

3.74 $$\begin{aligned} d^*= \sup _{d_1 \ge 0} \big \{\forall d \in [0, d_1): \, {\textbf {TW}}\text { holds for } M^-_d(i_{-\infty }) \big \} >0. \end{aligned}$$

Assume that $d^*$ does violate neither (3.69) nor (3.70). The manifold $M^-_{d^*}(i_{-\infty })$ exists locally around the fixed point $(a,a',i,i') = (0,0,i_{-\infty },0)$ due to Corollary 3.4. Let $R >0$. Since the System $S_{d^*}$ is a smooth vector field, also the continuation

3.75 $$\begin{aligned} M^-_{d^*}(i_{-\infty }) \cap \{ x \in \mathbb {R}^4: \vert \vert x \vert \vert _\infty < R\} \end{aligned}$$

is a smooth and bounded manifold. If it is non-negative, we proceed with the next paragraph. If not, choose a representation

$$\begin{aligned} M^-_{ d^*}(i_{-\infty },x) \vert _{x \in (- \infty ,T]} \end{aligned}$$

for some finite $T \in \mathbb {R}$, such that the manifold has become strictly negative at time *T*. By Proposition 3.6, there exists an open interval $I \subset \mathbb {R}^+_0, d^*\in I$, such that for all $d \in I$: the manifolds $M^-_{ d}(i_{-\infty },x) \vert _{x \in (- \infty ,T]}$ exists and are continuous in *d*. However, for all $d < d^*$, these manifolds are also non-negative, by choice of $d^*$. Thus, also $M^-_{ d^*}(i_{-\infty },x) \vert _{x \in (- \infty ,T]}$ must be non-negative.

Since $R>0$ was arbitrary, the entire manifold $M^-_{ d^*}(i_{-\infty })$ must be non-negative. Since it is not constant, it fulfills the conditions of Proposition 3.16, so

3.76 $$\begin{aligned} \lim _{x \rightarrow + \infty } i_{ d^*}(x) > 2 - i_{-\infty }- d^*\frac{2(r+1)}{c^2} \ge 0, \end{aligned}$$

in view of Condition (3.70), which was not violated. Now, Lemma 3.17 yields some structural results: it holds that $$i'_{ d^*}(x) < 0, a_{ d^*}(x) >0$$, and $$a_{ d^*}(x)$$ has a unique local and global maximum. Since $$a_{ d^*}(x), i_{ d^*}(x)$$ converge as $$x \rightarrow + \infty $$, there exists some $$x^*$$ as claimed in Lemma 3.14, which yields monotonicity of the tail. Thus, we have verified **TW** also for $$ d^*$$, and can again apply Proposition 3.8 to $$M^-_{ d^*}$$. This proves **TW** in a local neighborhood of $$ d^*$$. Insofar, $$ d^*$$ can not have been chosen as claimed, and either (3.69) or (3.70) must be violated for $$d^*$$. $$\square $$

By choosing $i_{-\infty }$ arbitrarily close to 1, Theorem 3.18 has a simple

##### Corollary 3.19

Let $r \ge 0, c \ge 2$. Then, for all $d \ge 0$ such that

3.77 $$\begin{aligned} d < \min \left\{ \frac{3c}{2} , \frac{c^2}{2(r+1)} \right\} , \end{aligned}$$

there exists some $i_{-\infty }\in (1,2)$ such that the manifold $M^-_d(i_{-\infty })$ is a non-negative traveling wave.

#### The invading front

It remains to show that there exists a wave where $i_{+\infty }= 0$, despite the $\mathcal {O}(d)$-estimate regarding the limits (3.73). We begin with the simple

##### Lemma 3.20

For all $d> 0, c>0, r \ge 0$, the manifold

3.78 $$\begin{aligned} M^-_d (2) \end{aligned}$$

is not non-negative.

##### Proof

Asymptotically as $x \rightarrow - \infty$: $a(x) >0$ by Corollary 3.4. Assume that $a(x),i(x) \ge 0$ for all $x \in \mathbb {R}$. Then, denoting the other limit of the wave as $i_{+\infty }= \lim _{x \rightarrow + \infty } i(x) \ge 0$, Proposition 3.16 implies

3.79 $$\begin{aligned} 2 + i_{+\infty }<2, \end{aligned}$$

a contradiction. $$\square $$

Given a non-negative and converging trajectory $M^-_d(K_0)$, the following lemma yields continuity and non-negativeness of $M^-_d(K)$ with respect to *K* in a neighborhood of $K_0$:

##### Lemma 3.21

Let $c \ge 2, d > 0, r \ge 0$ and $K_0 \in (1,2)$. Assume that the manifold $M^-_d(K_0)$ is a non-negative traveling wave, and thus converges as $x \rightarrow + \infty$, where

3.80 $$\begin{aligned} \lim _{ x \rightarrow + \infty } i(x) = i_{+\infty }\in (0,1). \end{aligned}$$

Then, there exists an open interval $I \subset (1,2), K_0 \in I$ such that for all $K \in I$: the entire manifold $M^-_d(K)\vert _{ x \in \mathbb {R}}$ is continuous in *K*, and moreover is also a non-negative traveling wave.

##### Proof

Consider $M^-_d(K_0)$ as proposed, and denote its limit as $i_{+\infty }^*$. We use Corollary 3.5 to control $M^-_d(K,x) \vert _{x \in ( - \infty , T]}$ for some $T \in \mathbb {R}$. By Corollary 3.3, the fixed point $(0,0,i_{+\infty }^*,0)$ is Lyapunov stable, and we can control also the tail as $x \rightarrow + \infty$.

Choose some

3.81 $$\begin{aligned} \rho \in (0, i_{+\infty }^*) . \end{aligned}$$

There exists $\epsilon >0$, such that for all trajectories $a(x), a'(x), i(x), i'(x)$ that start with in range of $\epsilon$ to $(0,0,i_{+\infty }^*,0)$:

3.82 $$\begin{aligned} \Big \vert \big ( a(x), a'(x), i(x), i'(x) \big ) - \big (0,0,i_{+\infty }^*,0 \big ) \Big \vert \le \rho , \quad \hbox { for all}\ x \ge 0, \end{aligned}$$

since the fixed point is Lyapunov-stable. Wlog let $\epsilon \le \rho$. There exists a finite phase-time *T*, such that

3.83 $$\begin{aligned} \Big \vert M^-_d(K_0,T) - \big (0,0,i_{+\infty },0 \big ) \Big \vert \le \frac{ \epsilon }{2}. \end{aligned}$$

We now use the continuity of $M^-_d(K,x) \vert _{ x \in (-\infty ,T]}$ with respect to *K*, see Corollary 3.5: there exists a $\delta >0$ with $K_0 - \delta > 1$, and such that for all $K \in [K_0 - \delta , K_0 + \delta ]$: the manifold $M^-_d(K,x) \vert _{ x \in (-\infty ,T]}$ is of distance at most $\epsilon / 2$ to $M^-_d(K_0,x) \vert _{ x \in (-\infty ,T]}$.

Then, for all $K \in [K_0 - \delta , K_0 + \delta ]$, the manifold $M^-_d(K_0,x) \vert _{ x \in (-\infty ,T]}$ is also strictly non-negative, since $i(x) \ge i_{+\infty }^*- \frac{\rho }{2} > 0$ for all $x \in (-\infty ,T]$, by Lemmas 3.9 and 3.11. Moreover, at time *T*, any such manifold has entered the $\rho$-neighborhood of $(0,0,i_{+\infty }^*,0)$. Thus, $i(x) \ge 0$ for all $x \in \mathbb {R}$ and we can apply Lemma 3.11 to conclude that also $a(x) \ge 0$ for all $x \in \mathbb {R}$. Moreover, all such trajectories have at most distance $\epsilon \le \rho$ from $M^-_d(K_0)$. Since we can choose $\rho$ as small as we want, this also proves continuity of the entire manifold $M^-_d(K,x) \vert _{ x \in \mathbb {R}}$ in *K*.

$$\square $$

Continuity of the traveling waves w.r.t. *K* implies the existence of an invading front:

##### Theorem 3.22

Let $c \ge 2, r \ge 0, d > 0$ and $K_0 \in (1,2)$ such that $M^-_d(K_0)$ is a non-negative traveling wave. Then, there exists also some $K_1 \in [K_0,2)$, such that $M^-_d(K_1)$ is an invading front.

##### Proof

We proof a sort of intermediate value theorem, increasing *K* as much as possible. For a manifold $M^-_d(K)$ that is non-negative and converges, we denote $i_{+\infty }(K):= \lim _{x \rightarrow + \infty } i(x) \ge 0$.

We are done if $i_{+\infty }(K_0) =0$, so we assume $i_{+\infty }(K_0) >0$ in the following. Then, the manifold $K^-_d(K_0)$ fulfills the conditions of Lemma 3.21. It follows that there exists a neighborhood $I \subset \mathbb {R}$ with $K_0 \in I$, such that the entire manifold $M^-_d(K)$ is continuous w.r.t. $K \in I$, including its limit $i_{+\infty }(K)$. We extend this to a non-local statement by defining

3.84 $$\begin{aligned} \begin{aligned} K_1 := \sup _{ L \ge K_0 } \Big \{ \forall K \in [K_0, L) \Big \vert \, M^-_d(K) \text { is non-negative and converges} \Big \}. \end{aligned} \end{aligned}$$

Recall that for $K=2$, the manifold $M^-_d(2)$ eventually becomes negative by Lemma 3.20, so it must hold that $K_0< K_1 < 2$. For all $K \in [K_0, K_1)$, the manifold $M^-_d(K)$ is non-negative and converges, by the definition of $K_1$. By Corollary 3.4, the manifold $M^-_d(K_1)$ exists locally around the fixed point. Since $S_d$ is a smooth vector field, also the continuation

3.85 $$\begin{aligned} M^-_d(K_1) \vert R := M^-_d(K_1) \cap \big \{ x \in \mathbb {R}^5: \, \vert \vert x\vert \vert _\infty < R \big \} \end{aligned}$$

exists and is a smooth manifold, for any $R >0$. Now, since $M^-_d(K)$ is non-negative and converges for all $K \in [K_0,K_1)$, also $M^-_d(K_1) \vert R$ must be non-negative, by a continuity argument completely analogue to that in the proof of Theorem 3.18. Since *R* can be chosen arbitrarily large, the entire manifold $M^-_d(K_1)$ is non-negative, and thus also converges.

We are done if $i_{+\infty }(K_1) = 0$. So let us assume that $i_{+\infty }(K_1) >0$. In this case, we again apply Lemma 3.21 to $M^-_d(K_1)$, resulting in the existence of non-negative and converging solutions for in an open neighborhood of $K_1$, a contradiction. $\square$

We finish the

##### Proof of Theorem 1.1

For *d* as claimed, Corollary 3.19 and Theorem 3.22 imply the existence of an invading front. It remains to determine its asymptotic behavior, therefore we consider only $d \in (0,1)$.

As $x \rightarrow - \infty$, the rate of convergence along the instable manifold is given in Corollary 3.4 (depending on $i_{-\infty }$). Regarding the behavior as $x \rightarrow + \infty$, we take a look at the linear Representation (3.10) and the Eigenvalues (3.7). For $d \in (0,1)$ and $i_{+\infty }\in [ 1 - \frac{c^2}{4},1)$, we can order the purely real-valued Eigenvalues of the limit:

3.86 $$\begin{aligned} 0>\lambda _4 \ge \lambda _3 > \lambda _2, \end{aligned}$$

where the inequalities are strict if $i_{+\infty }> 1 - \frac{c^2}{4}$. This ordering is crucial: even though we do not have a complete description of the asymptotics, we can determine the rate of convergence, as some components of the system converge with speed $\lambda _4$: we apply the same trapping argument as for $S_0$, see Proposition 6.3 in Kreten (2022). As long as $\lambda _4 \ge \lambda _3$, we know by an analysis of the phase space that the two components $a(x), a'(x)$ converge along the Eigenvector $e_4$ (3.7), associated to $\lambda _4$. We now must differentiate between the critical and the noncritical case.

For the noncritical case, where $i_{+\infty }\ne 1 - c^2/4$, the asymptotics are equivalent to the linear System (3.10). This implies that the system approaches its limit exponentially with rate $\lambda _4$.

The critical case $i_{+\infty }= 1 - c^2/4$ is a bifurcation point of the system: solutions that converge to $i_{+\infty }< 1- c^2$ spiral, since the Eigenvalues $\lambda _3$ and $\lambda _4$ have an imaginary part (3.7), they do not spiral and converge exponentially fast if the limit fulfills $i_{+\infty }> 1 - c^2/4$. Luckily, we do not have to find a complete representation for the behavior around the bifurcation point: we only need to determine the dynamics of a trajectory that converges to the specific limit $i_{+\infty }= 1 - c^2/4$. In this case, we still know that the center manifold of the fixed point $(a,a',i,i') = (0,0,i_{+\infty },0)$ has dimension one, and thereby must coincide with the adjacent line of fixed points. Thus, any trajectory that converges to $i_{+\infty }= 1 - c^2/4$ must be contained in the remaining three-dimensional strictly hyperbolic subsystem. The Eigenvalue $\lambda _3 = \lambda _4$ has algebraic multiplicity 2, but geometric multiplicity 1, see the Eigenvectors (3.7). Along the corresponding manifold, trajectories converge as fast as $x \cdot \exp ( - x \lambda _4)$, cf. chapter 9 in Boyce et al. (2017). $\square$

## Mathematical background and outlook

### Stability of FKPP waves

Reaction–diffusion systems provide the natural framework for the mathematical analysis of the spatiotemporal patterns that can arise from a set of local interaction rules, there exists an extensive literature that focuses on systems with a biological motivation (Britton 2003; Othmer et al. 2009; Volpert and Petrovskii 2009). Two works presumably constitute the foundation of the field. The first is Turings *The Chemical basis of Morphogenesis* (Turing 1952). The second, which is of importance for the present work, is the analysis of the FKPP-equation in the 1930s by Fisher (1937) and Kolmogorov et al. (1937). This equation describes the spreading of a fitter population, or “The Wave of Advance of Advantageous Genes” (Fisher 1937). In its simplest form and in one spatial dimension $z \in \mathbb {R}$, this equation describes the evolution over time of a density of diffusing particles $A(t,z) \ge 0$ that undergo dampened growth:

4.1 $$\begin{aligned} \partial _t A&= \partial _{z}^2 A + F(A), \end{aligned}$$

where $F:\mathbb {R} \rightarrow \mathbb {R}$ is a reaction that satisfies

4.2 $$\begin{aligned} F(0) = F(1) = 0, F'(0)&>0, F'(1) \le 0, F''(u) <0 \quad \forall u \in (0,1). \end{aligned}$$

**Fig. 4:**
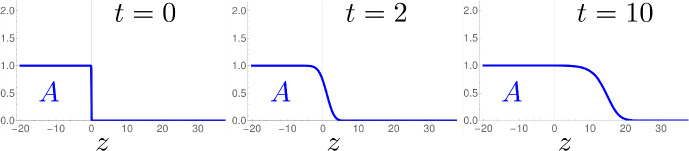
Simulation of the FKPP-Equation (4.1) with logistic growth, given Heaviside initial data $A(0,z) = \mathbb {1}_{z \le 0}$. The front converges to the traveling wave with speed $c_{\min }=2$, which is well-approximated already for $t=10$

The classical example for *F* is the logistic growth function $F(A) = A - A^2$. Non-negative traveling waves of System (4.1) are referred to as *pulled*, since their speed is uniquely determined by their asymptotic behavior at the right tail (considering right-traveling waves as depicted in Fig. 4).

For the FKPP-equation, several proofs show that the traveling waves are the global attractors of the system. If the PDE starts in Heaviside initial data, then its front converges to the critical traveling wave as $t \rightarrow \infty$ (Kolmogorov et al. 1937; Uchiyama 1977; Bramson 1983), as depicted in Fig. 4. Given that the initial data decays fast enough, then it is always the critical wave which arises in the large time limit. The classical convergence proofs rely on a comparison principle and on the fact that if the initial data is monotone, then the front remains monotone for all $t\ge 0$. Notably, Bramson (1983) used a Feynman–Kac formula to relate solutions of the FKPP-equation to branching Brownian motion, which yields precise estimates on both the speed and the decay of the front.

FKPP-related systems often show a similar behavior: They form heteroclinic fronts that connect an unstable state to a stable one and have a certain minimal speed (Ducrot et al. 2019; Faye and Holzer 2020). However, convergence results are sparse for systems with more than one reactant, and essentially rely on monotonicity and comparison techniques (Ducrot et al. 2019; Girardin and Lam 2019). In a model with two interacting and competing species, Bovier and Hartung used a Feynman–Kac formula to derive precise results on the spreading speeds (Bovier and Hartung 2022).

For the present System (1.1), a global convergence result is difficult to prove since the active particles form a non-monotone pulse, being accompanied by a monotone front of inactive particles of the very same speed. For systems that lack a comparison principle, typically only the stability against small perturbations can be proven, we recommend the monograph of Sandstede for an introduction to the field (Sandstede 2002). For noncritical fronts (those with a spectral gap of the linearized perturbation) and for bounded weights, Sattinger (1976) proved the first general result in 1976. general result (Bates and Jones 1989), which is also the basis for many further analyses. One of those is a recent study by Ghazaryan et al. (2013), who proved several general results in the case of unbounded weights, even for mixed ODE-PDE models, but also under the assumption of a spectral gap. They introduced the notion of *convective stability* – while Physicists refer to the same phenomenon as *convective instability* (Tobias et al. 1998). The result of Ghazaryan et al. (2013) can in principle be used to study the supercritical waves of System (1.1), even in the case $d=0$. A first numerical analysis for the case $d>0$ indicates that the discrete spectrum of the linearized perturbation is indeed stable in a convective setting similar to that in the present work. I expect these waves to be exponentially convectively stable. The lack of an appropriate bound on the discrete spectrum in the case $d=0$ (see Appendix B) hinders a numerical analysis.

Stability of the critical waves, which are the most important ones for FKPP systems, is much more intricate. For the case of a single reactant, the stability of critical fronts has been proven for general reaction-terms, by Gallay (1994). The author proved that the algebraic speed of decay $t^{-3/2}$ is indeed optimal, we expect this also to be true in the present case. For more than one reactant, rigorous proofs were sparse until recently.

Faye and Holzer (2020) could prove the local stability of the critical waves of a Lokta–Volterra system of two species. While their proof is restricted to a particular category of systems of two reactants, it shows how the underlying technique—that goes back to Zumbrun and Howard (1998)—can be used to study systems with more than one reactant. The computations are quite demanding: a precise analysis of the Evans-function in the neighborhood of the essential spectrum is required, involving several changes of coordinates while keeping track of the resulting error terms. I want to thank the authors for remarking that their proof is largely independent of the actual weight of the particular system, which initiated the idea for this project.

Very recently, Avery and Scheel extended this result to arbitrary critical pulled fronts of multi-reagent systems (Avery and Scheel 2021, 2022; Avery 2024). Additionally, these results show that the perturbations can induce a logarithmic shift of the front, under slightly weaker assumptions regarding the initial perturbations when compared to the setting of Faye and Holzer (2020). This logarithmic shift has long been known for the scalar case (Bramson 1983), and essentially carries over to the multi-reagent case.

We expect that Theorem 1.2 also holds if the weight at the back of the front is integrable with algebraic decay, since the back of the system is diffusively stable. However, controlling the nonlinear terms is challenging in this case, typically this again requires the use of an exponential weight (Avery et al. 2023), though we strongly believe that it is not necessary in the present case.

### Convective stability

Proving convective stability requires the use of an unbounded weight. Controlling the nonlinearity in a space with an unbounded weight requires some detours, typically involving and intertwining estimates on both the weighted perturbations, and the evolution of the unweighted full PDE. There are typically three steps involved: First, find a suitable open set of functions $E \subset \mathcal {B}$ such that we can control the nonlinear perturbation $\tilde{Y}(t)$ as long as $\tilde{Y}(t) \in E$, and choose initial data under which $\tilde{Y}(t) \in E$, for some positive time $t \in [0, T^*)$, where $T^*$ is the first exit time of *E*. Second, prove that the weighted perturbation $u(t) = \tilde{Y}(t)/w$ decays while $\tilde{Y}(t) \in E$. Third, use the decay of *u*(*t*) to prove that $T^*= + \infty$, i.e. we can control the nonlinearity $\tilde{Y}(t) \in E$ for all $t \ge 0$.

The first rigorous proof of convectively stability is probably due to Pego and Weinstein (1994), who proved convective stability of solitary traveling waves – locally concentrated waves in fluids that can pass each other without interaction – of the Korteweg-de Vries equation. Pego and Weinstein control the weighted nonlinearity, which is of order $\mathcal {O}(u^2w)$ for the weight $w(x) = e^{- \alpha x}$, via the estimate

4.3 $$\begin{aligned} \vert \vert u^2 e^{- \alpha x} \vert \vert _{H^1} \le \vert \vert e^{-\alpha x} u \vert \vert _{H^1} \cdot \vert \vert u \vert \vert _{H^1} = \vert \vert \tilde{Y} \vert \vert _{H^1} \cdot \vert \vert u \vert \vert _{H^1}. \end{aligned}$$

In words: The weighted quadratic term $u^2w$ can be treated like a linear one as long as the unweighted perturbation $uw =\tilde{Y}$ remains bounded. The term $\vert \vert \tilde{Y} \vert \vert _{H^1}$ is controlled via energy methods that are tailored precisely to the underlying PDE. While the general idea of this argument is quite intuitive, the detailed execution is lengthy and rather complicated (Pego and Weinstein 1994).

Ghazaryan (2009) proved convective stability of a traveling wave of a reaction–diffusion system whose structure is quite similar to that of System (1.1). This PDE is a model for a combustion front without heat-loss, in one spatial dimension it reads

4.4 $$\begin{aligned} \begin{aligned} \partial _t Y_1&= \partial _{z}^2 Y_1 + Y_2 f(Y_1), \\ \partial _t Y_2&= \epsilon \partial _{z}^2 Y_2 - \kappa Y_2 f(Y_1), \end{aligned} \end{aligned}$$

where $Y_1(t,z) \ge 0$ is the temperature and $Y_2(t,z) \ge 0$ is the concentration of a viscous fuel, with burning reaction

4.5 $$\begin{aligned} f(Y_1) = e^{ - 1/Y_1 } \mathbb {1}_{ \{ Y_1 \ge 0 \} }, \end{aligned}$$

for $0 < \epsilon \ll 1, \kappa >0$. This model has a right-traveling wave solution that invades into the region with fuel, and burns it all while reaching a limiting temperature. As a result, the left limit of the wave is a non-hyperbolic equilibrium (there is no reaction in absence of fuel). This implies that the linearized perturbation is only spectrally stable with spectral gap if one uses a convective weight – the situation is the same as for the present System (1.1). Ghazaryan proves that this wave is convectively stable (we ignore the shift along the wave in the following presentation), by exploiting the different roles of the temperature $Y_1$ and the fuel $Y_2$.

Using the explicit structure of the reaction–diffusion PDE (4.4), Ghazaryan is able to get an a-priori bound on the temperature: $\vert \vert Y_1 \vert \vert _{BC^1} \le M$. Using both weighted and unweighted terms, the nonlinear reaction can be rewritten as

4.6 $$\begin{aligned} u_2 (t,x) \exp \Big ( - \frac{1}{Y_1}\Big ), \end{aligned}$$

which leads to an estimate of type

4.7 $$\begin{aligned} \vert \vert u_2 \exp \Big ( - \frac{ 1}{Y_1} \Big ) \vert \vert _{H^1} \le L \cdot \vert \vert u_2 \vert \vert _{H^1}. \end{aligned}$$

Again, the nonlinearity can be treated like a linearity in $u_2$, as long as the a-priori estimate (4.7) holds. Estimate (4.7) was indeed a blueprint for the present work, which takes a very similar route. However, the detailed estimate in Ghazaryan (2009) is much more difficult.

Recently, Garenaux could prove the convective stability of a critical pulled front that undergoes a Turing bifurcation at its back and thereby becomes unstable (Garenaux 2024). The employed technique, that shows that the dynamics at the back are approximated by a Ginzburg–Landau equation, requires the use of mode-filters in an appropriate Fourier space. This is technically quite demanding, contrasting the present Feynman–Kac estimate.

## Figures and Tables

**Fig. 5:**
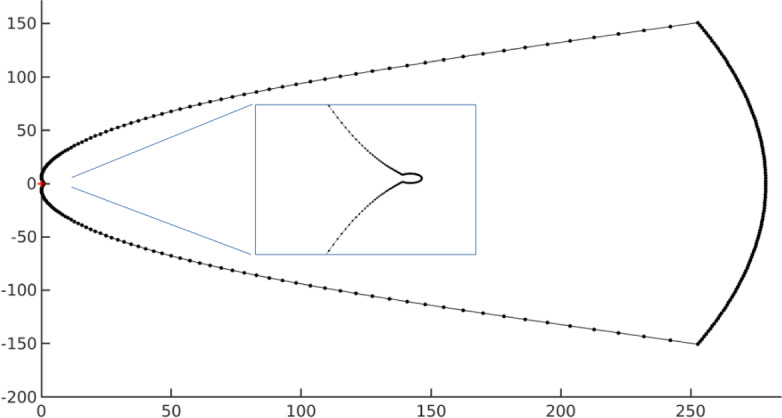
The Evans function for the weight $w(x) = e^{-x}$, for $c=2.0, d = 0.3$ and $r=1$, evaluated along the boundary of the domain $\mathcal {P}$, see (B26). The boundary consists of three parts: a semicircle with radius $\delta = 10^{-3}$ around the origin, two lines along the imaginary axis, and a semi-circle with radius *R*, see Proposition B.2. The winding number is equal to zero, as a consequence there are no elements of the discrete spectrum of $\mathcal {L}$ within $\mathcal {P}$. The simulations for all other parameters result in similar pictures

## The essential spectrum given a convective weight

In this section, we analyze the essential spectrum of the linear operator $\mathcal {L}$, as defined by (2.5), and prove Proposition 2.2. For $\lambda \in \mathbb {C}$, consider the Eigenvalue problem $$\lambda \cdot \begin{pmatrix}u \\ v \end{pmatrix} = \mathcal {L} \begin{pmatrix}u \\ v \end{pmatrix}$$. With the help of the auxiliary variables

A1 $$\begin{aligned} U := \begin{pmatrix} u \\ u' \\ v \\ v' \end{pmatrix}, \end{aligned}$$

the Eigenvalue problem can be rewritten as an equivalent linear system of ODEs:

A2 $$\begin{aligned} U' = M(x,\lambda ) \cdot U, \end{aligned}$$

for a matrix $M(x,\lambda )$ given by

A3 $$\begin{aligned} M(x,\lambda )&:= \begin{pmatrix} 0 & \quad 1 & \quad 0 & \quad 0 \\ \lambda + \xi _u(x) & \quad -(c + 2\frac{w'(x)}{w(x)}) & \quad a(x) & \quad 0 \\ 0 & \quad 0 & \quad 0 & \quad 1 \\ -\frac{2a(x)+i(x)+r}{d} & \quad 0 & \quad \frac{\lambda + \xi _v(x)}{d} & \quad - \frac{c+2d \frac{w'(x)}{w(x)} }{d} \end{pmatrix}, \end{aligned}$$

A4 $$\begin{aligned} \xi _u(x)&:= 2a(x)+i(x)-1 -c \frac{w'(x)}{w} - \frac{w''(x)}{w(x)}, \end{aligned}$$

A5 $$\begin{aligned} \xi _v(x)&:= -a(x) - c \frac{w'(x)}{w(x)} - d \frac{w''(x)}{w(x)}. \end{aligned}$$

We use this representation for the

### Proof of Proposition 2.2

For any non-negative traveling wave, *a*(*x*) vanishes as $x \rightarrow \pm \infty$, and *i*(*x*) converges. Moreover, both converge exponentially fast at both ends. The same applies to the matrices $M(x,\lambda )$, which we call exponentially localized. If this is given, the essential spectrum of $\mathcal {L}$ can be bounded: the Fredholm borders of $\mathcal {L}$ are given by those values of $\lambda \in \mathbb {C}$, for which one of the limit matrices $M(\pm \infty , \lambda )$ is defect. – *Remark: The remaining discrete spectrum to the right of these sets is analyzed in Appendix* B. – Denoting as $\mathcal {E}(M)$ the Eigenvalues of a matrix *M*, the Fredholm borders of $\mathcal {L}$ are given by

A6 $$\begin{aligned} \begin{aligned} \Sigma _{Fred} =&\, \Big \{ \lambda \in \mathbb {C} \Big \vert \, \exists \, \mu \in \mathcal {E}\big ( M(+\infty , \lambda ) \big ): \, \mathfrak {Re}(\mu ) = 0 \Big \} \\ \cup&\, \Big \{ \lambda \in \mathbb {C} \Big \vert \, \exists \, \mu \in \mathcal {E}\big ( M(-\infty , \lambda ) \big ): \, \mathfrak {Re}(\mu ) = 0 \Big \}. \end{aligned} \end{aligned}$$

For more details, we recommend the monograph by Sandstede (2002), or the specific analysis of a similar system in section 2 of Faye and Holzer (2020). We focus on the critical invading fronts, but in principle, the following results can be applied to the other critical traveling waves of the system (with minimal speed depending on the chosen limits).

For $d\in (0,1)$, consider now an invading front *a*(*x*), *i*(*x*) with speed $c = 2$. We first analyze the matrix $M(+ \infty , \lambda )$. Given that $i_{+\infty }=0$ and with Weight (2.23), the limit of $M(x,\lambda )$ (A3) at $x = + \infty$ is given by

A7 $$\begin{aligned} \begin{pmatrix} 0 & \quad 1 & \quad 0 & \quad 0 \\ \lambda -1 + 2 \alpha _+ - \alpha _+^2 & \quad -2 +2 \alpha _+ & \quad 0 & \quad 0 \\ 0 & \quad 0 & \quad 0 & \quad 1 \\ -\frac{r}{d} & \quad 0 & \quad \frac{\lambda + 2 \alpha _+ - d\alpha _+^2 }{d} & \quad - \frac{2 - 2d \alpha _+ }{d} \end{pmatrix}. \end{aligned}$$

This matrix has two pairs of Eigenvalues:

A8 $$\begin{aligned} \nu _\pm (\lambda , \alpha _+)&= -1 \pm \sqrt{i_{+\infty }+ \lambda } + \alpha _+, \end{aligned}$$

A9 $$\begin{aligned} \eta _\pm (\lambda , \alpha _+)&= \frac{1}{d} \big ( - 1 \pm \sqrt{d\lambda +1} \big ) + \alpha _+. \end{aligned}$$

We define the set

A10 $$\begin{aligned} \Sigma _{\nu }&:= \big \{ \lambda \in \mathbb {C} \big \vert \, \mathfrak {Re} \big ( \nu _+(\lambda ) \big ) = 0 \big \}. \end{aligned}$$

For $w \equiv 1$ (if $\alpha _+ = 0$), the set $\Sigma _{\nu }$ is a parabola that goes into the right half-plane, as depicted in Fig. 2. In order to stabilize it, we need to operate in a space with weight

A11 $$\begin{aligned} w(x) = e^{ - \frac{c}{2} x} = e^{-x}, \qquad \forall x \ge 1. \end{aligned}$$

This is the only possible choice for stabilizing the front of a critical FKPP-wave, a classical result, check (Sattinger 1976; Faye and Holzer 2018). For $\alpha _+ = 1$, the Eigenvalues of $M(+ \infty , \lambda )$ are then given by

A12 $$\begin{aligned} \nu _\pm (\lambda ,1) = \pm \sqrt{\lambda }, \quad \eta _\pm (\lambda ,1) = \frac{1}{d} \Big ( d-1 \pm \sqrt{ d \lambda +1 } \Big ). \end{aligned}$$

The pair $\nu _\pm$ creates an essential spectrum that lies on the negative real axis and goes up to the origin:

A13 $$\begin{aligned} \Sigma _{+}&:= \Sigma _\nu = \Big \{ \lambda \in \mathbb {C} \big \vert \, \mathfrak {Re}\lambda \le 0, \mathfrak {Im} \lambda = 0 \Big \}. \end{aligned}$$

Since $d \in (0,1)$, the Eigenvalue $\eta _-(\lambda )$ does not touch the imaginary axis, only the Eigenvalue $\eta _+(\lambda )$ crosses the imaginary axis along a parabola that lies in the strict negative half-plane:

A14 $$\begin{aligned} \Sigma _2&:= \Sigma _{\eta } = \Big \{ \lambda \in \mathbb {C} \big \vert \, \mathfrak {Re}\lambda = \frac{1}{d} \big ( 2 (1-d)^2 -1 - \vert d\lambda +1 \vert ^2 \big ) \Big \}. \end{aligned}$$

We now consider the other limit, $M(- \infty , \lambda )$, given by

A15 $$\begin{aligned} \begin{pmatrix} 0 & 1 & 0 & 0 \\ \lambda + i_{-\infty }-1 + 2 \alpha _- - \alpha _-^2 & -2 +2 \alpha _- & 0 & 0 \\ 0 & 0 & 0 & 1 \\ -\frac{r+i_{-\infty }}{d} & 0 & \frac{\lambda + 2 \alpha _- - d\alpha _-^2 }{d} & - \frac{2 - 2d \alpha _- }{d} \end{pmatrix}, \end{aligned}$$

for some $i_{-\infty }\in (1,2)$, and Eigenvalues

A16 $$\begin{aligned} \sigma _\pm (\lambda , \alpha _-)&= -1 \pm \sqrt{i_{-\infty }+ \lambda } + \alpha _-, \end{aligned}$$

A17 $$\begin{aligned} \phi _\pm (\lambda , \alpha _-)&= \frac{1}{d} \big ( - 1 \pm \sqrt{d\lambda +1} \big ) + \alpha _-. \end{aligned}$$

In order to stabilize $\phi _+$ away from the origin, we can pick any $\alpha _- >0$, leading to the parabola

A18 $$\begin{aligned} \Sigma _-&:= \Sigma _\phi = \Big \{ \lambda \in \mathbb {C} \big \vert \, \mathfrak {Re}\lambda = \frac{1}{d} \big ( 2(1-d\alpha _-)^2 - 1 - \vert \lambda +1 \vert \big ) \Big \}. \end{aligned}$$

As long as $\alpha _- < 2 / d$, the Eigenvalue $\phi _-$ does not touch the imaginary axis. The Eigenvalue $\sigma _+$ crosses the imaginary axis along a parabola that lies in the strict negative half-plane:

A19 $$\begin{aligned} \Sigma _3&:= \Sigma _{\sigma } = \Big \{ \lambda \in \mathbb {C} \big \vert \, \mathfrak {Re}\lambda = - i_{-\infty }- 2(1-\alpha _-)^2 - \vert i_{-\infty }+ \lambda \vert \Big \}. \end{aligned}$$

Lastly, we check that we do not overstabilize $\sigma _-$. It holds that

A20 $$\begin{aligned} \mathfrak {Re} \big ( \sigma _-(\lambda ) \big ) = 0 \, \Leftrightarrow \, \alpha _- -1 = \mathfrak {Re} \big ( +\sqrt{ i_{-\infty }+ \lambda } \big ). \end{aligned}$$

The set $\{ \mathfrak {Re} \big ( \sigma _-(\lambda ) \big ) = 0 \}$ is obviously empty as long as

A21 $$\begin{aligned} \alpha _- < 1. \end{aligned}$$

$$\square $$

## Numerical evaluation of the discrete spectrum

We present a numerical analysis of the discrete spectrum of $\mathcal {L}$ as in Proposition 2.2. For this, we consider only a single type of weight, namely

B1 $$\begin{aligned} w(x) = e^{-x}. \end{aligned}$$

This choice allows for an efficient energy based estimate regarding the maximal possible size of an Eigenvalue with non-negative real-part, presented in Sect. B.1. In the subsequent Sect. B.2, we briefly review the theory of the Evans function and its numerical evaluation via STABLAB, a tool by Barker et al. (2015). The computations show that $\mathcal {L}$ is spectrally stable in $\mathcal {H}^2$.

Moreover, if the non-negative discrete spectrum for the Weight (B1) is empty, then the same is also true for all

B2 $$\begin{aligned} w(x)_{\vert x \le -1} = e^{-\alpha _-x}, \quad \alpha _- < 1. \end{aligned}$$

As long as both limit matrices $M(\pm \infty , \lambda )$ are non-degenerate, then $\lambda$ is an element of the discrete spectrum of $\mathcal {L}$ as an operator on $BUC^2$ only if the same is true in $\mathcal {H}^2$. The reason for this is an exponential dichotomy: all (bounded or unbounded) eigenfunctions approach the eigenspaces of the limit matrices asymptotically, and thus either grow or decay exponentially fast. For more details, check chapter 3.2 in Sandstede (2002). Thus, $\mathcal {L}$ is also spectrally stable in *BUC*.

Note that the stability of the point spectrum of $\mathcal {L}$ also induces the following, slightly stronger statement: there exist $\delta _0, \delta _1 >0$, such that for the region

B3 $$\begin{aligned}&\Omega := \Big \{ \lambda \in \mathbb {C} \big \vert \, \mathfrak {Re} \lambda \ge - \delta _0 - \delta _1 \cdot \vert \mathfrak {Im} \lambda \vert \Big \} \end{aligned}$$

it holds that

B4 $$\begin{aligned}&\Omega \cap \Sigma _{d} = \emptyset . \end{aligned}$$

To conclude that Assumption B4 holds, see e.g. Proposition 2.2 in Alexander et al. (1990), which proves that there is a set $\Omega _1$ as in (B3), that contains only finitely many elements of the discrete spectrum of $\mathcal {L}$. By eventually choosing smaller $\delta _0, \delta _1$, it can easily be seen that there exists a set $\Omega \subset \Omega _1$ as claimed.

### An energy bound

The below idea is probably due to J. Humpherys, who also presents a refined version in Hendricks et al. (2015). A similar bound is found by Varas and Vega (2002), but their calculation turns out to be wrong due to a sign error. Consider the linear problem

B5 $$\begin{aligned} \lambda \cdot u_i(x) = D_i \cdot u_i''(x) + c_i \cdot u_i'(x) + \sum _{j=1}^n f_{i,j}(x) \cdot u_j(x), &  i = 1, \cdots , n, \end{aligned}$$

for $\lambda \in \mathbb {C}$, constants $D_i \ge 0, c_i \ge 0$, and functions $f_{i,j} \in L^\infty (\mathbb {R})$.

Now assume that there exists an Eigenvalue $\lambda \in \mathbb {C}$ with $\mathfrak {Re} \gamma \ge 0$, and associated eigenfunctions $u_i \in \mathcal {H}^2(\mathbb {R})$ (the *unweighted* Sobolev-space). The product structure of $\mathcal {H}^2$ induces an energy estimate on $\vert \lambda \vert$:

#### Theorem B.1

(An a priori bound for the positive discrete spectrum) Let $\lambda \in \mathbb {C}$ with $\mathfrak {Re} \lambda \ge 0$ and functions $\big ( u_i \in \mathcal {H}^2(\mathbb {R}) \big )_{i = 1 \cdots n}$ be a solution of the Eigenvalue-problem (B5). For $i = 1, \dots , n$, define

B6 $$\begin{aligned} M_i := \sum _{j=1}^n \vert \vert f_{i,j} \vert \vert _\infty . \end{aligned}$$

Then, the real-part of $\lambda$ is bounded:

B7 $$\begin{aligned} \mathfrak {Re} \lambda&\le \max _i \{ M_i \}. \end{aligned}$$

Moreover, if $D_i>0$ for all $i = 1, \dots , n$, such that the system is with non-degenerate diffusion, it holds that

B8 $$\begin{aligned} \vert \mathfrak {Im} \lambda \vert&\le \max _i \left\{ c_i \cdot \sqrt{ \frac{M_i}{D_i} } + M_i \right\} . \end{aligned}$$

#### Proof

In the following, the norm $\vert \vert . \vert \vert _2$ will be the $L^2$-norm with corresponding scalar product $\langle \,. \, \rangle$. Before proving the above statement, let us note that for any function $u \in \mathcal {H}^2(\mathbb {R})$, the product $\langle u', \bar{u} \rangle$ has no real-part, since

B9 $$\begin{aligned} \begin{aligned} \mathfrak {Re} \, \int _{\mathbb {R}} u'(x) \cdot \bar{u}(x) \, dx&= \frac{1}{2} \Big ( \int _{\mathbb {R}} u'(x) \cdot \bar{u}(x) + \bar{u}' \cdot u(x) \, dz \Big ) \\ &= \frac{1}{2} \int _{\mathbb {R}} \vert u(x)^2 \vert ' \, dx = 0. \end{aligned} \end{aligned}$$

Moreover, it holds by integration by parts that

B10 $$\begin{aligned} \langle u'', \bar{u} \rangle = - \vert \vert u' \vert \vert _2 ^2 \le 0. \end{aligned}$$

Now, assume that there exists an Eigenvalue $\lambda \in \mathbb {C}$ with $\mathfrak {Re} \lambda \ge 0$ and associated eigenfunctions $\big ( u_i \in \mathcal {H}^2(\mathbb {R}) \big )_{i = 1 \cdots n}$ that solve (B5). For for each $i \in 1 \cdots n$, multiply (B5) by $\bar{u}_i$:

B11 $$\begin{aligned} \lambda \cdot \vert \vert u_i \vert \vert _2 ^2&= D_i \cdot \langle u_i'', \bar{u_i} \rangle + c_i \cdot \langle u_i', \bar{u_i} \rangle + \sum _{j=1}^n \langle f_{i,j} \cdot u_j, \bar{u_i} \rangle \nonumber \\&= - D_i \vert \vert u_i' \vert \vert _2 ^2 + c_i \cdot \langle u_i', \bar{u_i} \rangle + \sum _{j=1}^n \langle f_{i,j} \cdot u_j, \bar{u_i} \rangle . \end{aligned}$$

Choose $k \in 1 \cdots n$ such that $\vert \vert u_k \vert \vert _2 = \max _i \vert \vert u_i \vert \vert _2$. By Eq. (B11), and since $\langle u_i', \bar{u_i} \rangle$ has no real-part:

B12 $$\begin{aligned} \begin{aligned} 0 \le \mathfrak {Re} \lambda \cdot \vert \vert u_k \vert \vert _2 ^2&= - D_k \cdot \vert \vert u_k' \vert \vert _2 ^2 + \sum _{j=1}^n \mathfrak {Re} \langle f_{k,j} \cdot u_j, \bar{u_k} \rangle \\&\le \vert \vert u_k \vert \vert _2 ^2 \cdot \sum _{j=1}^n \vert \vert f_{k,j} \vert \vert _\infty . \end{aligned} \end{aligned}$$

The claimed bound for $\mathfrak {Re} \lambda$ follows. We proceed similarly for the imaginary part of Eq. (B11):

B13 $$\begin{aligned} \mathfrak {Im} \lambda \cdot \vert \vert u_i \vert \vert _2 ^2&= c_i \cdot \langle u_i',\bar{u}_i \rangle + \sum _{j=1}^n \mathfrak {Im} \langle f_{i,j} \cdot u_j, \bar{u}_i \rangle . \end{aligned}$$

Again, for $$ \vert \vert u_k \vert \vert _2 = \max _i \vert \vert u_i \vert \vert _2 $$:

B14 $$\begin{aligned} \vert \mathfrak {Im}\lambda \vert \cdot \vert \vert u_k \vert \vert _2 ^2&\le c_k \cdot \vert \langle u_k',\bar{u}_k \rangle \vert + \sum _{j=1}^n \vert \vert f_{k,j} \vert \vert _\infty \cdot \vert \vert u_k \vert \vert _2 ^2. \end{aligned}$$

We are done if we can find a suitable bound for $\vert \vert u'_k \vert \vert _2$. This is where we require that $D_i>0$ for all $i \in 1 \cdots n$. Again take the real-part in Eq. (B11). The inequality $\mathfrak {Re} \lambda \ge 0$ implies that for all $i=1, \dots , n$:

B15 $$\begin{aligned} D_i \cdot \vert \vert u_i' \vert \vert _2 ^2&\le \sum _{j=1}^n \vert \vert f_{i,j} \vert \vert _\infty \cdot \vert \vert u_i \vert \vert _2 \cdot \vert \vert u_j \vert \vert _2 , \end{aligned}$$

and thus for *k* chosen as above: $\vert \vert u_k' \vert \vert _2 \le \sqrt{ \frac{M_k}{D_k} } \cdot \vert \vert u_k \vert \vert _2$. We use this in Eq. (B14) to bound

B16 $$\begin{aligned} \vert \mathfrak {Im} \lambda \vert \le c \cdot \sqrt{ \frac{M_k}{D_k} } + M_k. \end{aligned}$$

$$\square $$

Typically, Theorem B.1 yields a much better bound than the classical bounds that are based on the asymptotic behavior of the Evans function as $\vert \lambda \vert \rightarrow \infty$, cf. Section 4.2.2. in Sandstede (2002) for the idea, but is restricted to diffusive systems.

We apply Theorem B.1 to the Operator (2.5) that already encodes the transformation into the weighted space. For a traveling wave *a*(*x*), *i*(*x*) with speed $c=2$ and the weight $w(x) = e^{-x}$, $\mathcal {L}$ reduces to

B17 $$\begin{aligned} \begin{aligned} \mathcal {L}u&:= u_{xx} - u (2a+i ) -va, \\ \mathcal {L}v&:= d v_{xx} + v_x \cdot ( 2 - 2d ) + v \cdot ( a + d -2 ) + u( 2a+i+r). \end{aligned} \end{aligned}$$

Here, it is essential that $w'/w=-1$ is constant, such that we are in the setting of Theorem B.1. The following is a direct corollary:

#### Proposition B.2

For $d \in (0,1)$, consider $\mathcal {L}$ given by (B17), as an operator

B18 $$\begin{aligned} \mathcal {H}^2(\mathbb {R}) \times \mathcal {H}^2(\mathbb {R}) \rightarrow L^2(\mathbb {R}) \times L^2(\mathbb {R}). \end{aligned}$$

For all $\lambda \in \mathbb {C}$ in the discrete spectrum of $\mathcal {L}$ with $\mathfrak {Re}\lambda \ge 0$, it holds that

B19 $$\begin{aligned} \vert \lambda \vert&\le \sqrt{2} \left[ (2-2d) \sqrt{ \frac{5+r}{d} } + 5+r \right] . \end{aligned}$$

#### Proof

This is a direct consequence of Theorem B.1. We can estimate the two constants $M_a$ and $M_i$, see Eq. (B6), via the simple bounds $\vert i \vert \le 2, \vert a \vert \le 1$:

B20 $$\begin{aligned} M_a&= \vert 2a-i \vert _\infty + \vert a \vert _\infty \le 3, \end{aligned}$$

B21 $$\begin{aligned} M_i&= \vert a+d-2 \vert _\infty + \vert 2a+i+r \vert _\infty \le 5+r . \end{aligned}$$

$$\square $$

### The Evans function

The discrete spectrum within the region of consistent splitting of an operator can be evaluated numerically with the help of the Evans function. The following concepts are presented in much more detail by B. Sandstede, who wrote a great overview of the topic (Sandstede 2002), a more practical introduction and the numerical tool STABLAB (Barker et al. 2015) have been written by Barker et al. (2018).

Recall that for $\mathcal {L}$ as in Proposition 2.2, if $\lambda \in \Sigma _d$, this is equivalent to saying that there exists a bounded solution of a linear ODE of type

B22 $$\begin{aligned} U' = M(x,\lambda ) \cdot U, \qquad x \in \mathbb {R}, \end{aligned}$$

compare (A2). The matrix $M(x, \lambda )$ is given by (A3) and encodes the shift of the equation into the weighted space. It is now essential that we consider only $\lambda \in \mathbb {C}$ such that the matrices $M(x, \lambda )$ are analytic in $(\lambda ,x)$ up to $x = \pm \infty$, with strictly hyperbolic limits $M(\pm \infty , \lambda )$.

Since the limiting matrices $M(\pm \infty , \lambda )$ are hyperbolic, the theory of *exponential dichotomies* implies that any bounded solution *U*(*x*) must vanish exponentially fast as $x \rightarrow \pm \infty$. Moreover, it asymptotically approaches the unstable manifold of the constant matrix $M(- \infty , \lambda )$ as $x \rightarrow - \infty$, and the stable manifold of $M(+ \infty , \lambda )$ as $x \rightarrow + \infty$. Therefore, a bounded solution exists if and only if the trajectories that emerge from these manifolds intersect. This allows us to compute the Evans function: it is a determinant that evaluates to zero if and only if the solutions that decay at $-\infty$ and those that decay at $+\infty$ intersect, and are linearly dependent. The details are given in Section 4.1 in Sandstede (2002).

#### Definition B.3

(*Evans function*) Consider the ODE (B22), with $\lambda \in \mathbb {C}$ such that the matrices $M(x, \lambda )$ are analytic in $(\lambda ,x)$ up to $x = \pm \infty$, with hyperbolic limits $M(\pm \infty , \lambda )$.

Let

B23 $$\begin{aligned}&X_1(x, \lambda ), \cdots , X_{k_1}(x, \lambda ) \end{aligned}$$

be $k_1$ linearly independent representatives of those solutions that decay exponentially as $x \rightarrow - \infty$, and let

B24 $$\begin{aligned}&Y_1(x,\lambda ), \cdots , Y_{k_2}(x,\lambda ) \end{aligned}$$

be $k_2$ linearly independent representatives of those solutions that decay exponentially as $x \rightarrow + \infty$, with $k_1, k_2 >0$ and $k_1 + k_2 = n$. The Evans function $E(\lambda )$ is defined as

B25 $$\begin{aligned} E( \lambda ) := \text {det} \big ( X_1(x, \lambda ) \big \vert \cdots \big \vert X_{k_1}(x,\lambda ) \big \vert Y_1(x, \lambda ) \big \vert \cdots Y_{k_2, \lambda } \big ) \Big \vert _{x=0}. \end{aligned}$$

*Remark*: The Evans function is not unique, since the representatives $X_i, Y_i$ are not unique. However, it holds that $E(\lambda ) = 0$ if and only if System (B22) has a bounded solution. Moreover, $E(\lambda )$ is analytic if the $X_i, Y_i$ are chosen analytically in $\lambda$, which can be achieved, see section 4.1 in Sandstede (2002). Then, it suffices to calculate $E(\lambda )$ along the boundary of a domain $\mathcal {P} \subset \mathbb {C}$: the winding number of $E(\lambda )$ along $\partial \mathcal {P}$ corresponds to the number of zeros inside the domain.

We first need a numerical solution of a wave-profile, which goes hand in hand with finding the correct value of $i_{-\infty }$ such that the the other limit of the traveling wave fulfills $i_{+\infty }= 0$. Given some $i_{-\infty }>1$, we solve the forward ODE (3.5) under initial data that approximate the unstable manifold of a fixed point $(a,a',i,i') = (0,0,i_{-\infty },0)$, its asymptotic direction is given in Corollary 3.4. The limit as $x \rightarrow + \infty$ of this trajectory is Lyapunov-stable, such that the numerical forward solution converges if the initial data are sufficiently close to the unstable manifold of $(0,0,i_{-\infty },0)$. We then change $i_{-\infty }$ until $i_{+\infty }=0$. This differs from the approach suggested in Barker et al. (2018): the continuum of fixed points makes the proposed projective boundary value approach invalid. However, since the trajectory converges along the correct stable manifold as $x \rightarrow + \infty$, our forward approach is reasonable. This is a consequence of the trapping argument in Proposition 3.10, see also Proposition 6.3 in Kreten (2022). The numerical evaluation suggests that the value $i_{+\infty }$ is a monotone function of $i_{-\infty }$, such that any root-finding algorithm converges quickly to the (presumably) unique value of $i_{+\infty }$ such that $i_{-\infty }= 0$. The results are depicted in Table 1.

**Table 1:** The value of $\lim _{x \rightarrow - \infty }i(x)$ of the invading fronts (where $i_{+\infty }= 0$), for different values of *r*, *d* and for speed $c=2$

	$$d = 0.001 $$	$$d = 0.01 $$	$$d = 0.1 $$	$$d = 0.2 $$	$$d = 0.3 $$	$$d = 0.4 $$
$r=0$	1.99985	1.99848	1.98489	1.96999	1.95532	1.94091
$r=1$	1.99984	1.99842	1.98430	1.96897	1.95403	1.93948

The numerical values suggest that the effect of *d* is negligible, and that our bound in Lemma 3.16 is not sharp. The root finding was performed with Wolfram Mathematica

The various numerical challenges that arise when computing the Evans function, as well as their solutions, are described in detail by Barker et al. (2018), who also suggest using their library STABLAB (Barker et al. 2015). Given a numerical solution of the traveling front on an interval $x \in [-L,+L]$ (we choose $L = 50$), centered such that $a'(0) = 0$, STABLAB performs three main steps: first, the unstable and stable spaces of System (B22) are approximated by the unstable and stable eigenspaces of the constant matrices $M(\pm \infty , \lambda )$. Second, bases of these eigenspaces that are analytic in $\lambda$ are chosen. Third, the resulting ODEs are solved (forwards on $[-L,0]$ and backwards on [0, *L*]), in an exponentially weighted space to reduce computation time and increase numerical stability. We evaluate the Evans function along $\partial \mathcal {P}$, the boundary of the domain

B26 $$\begin{aligned} \mathcal {P} := \{ \gamma \in \mathbb {C}\big \vert \, \mathfrak {Re} \gamma \ge 0, \, \delta \le \vert \gamma \vert \le R \}, \end{aligned}$$

where the radius *R* is the upper bound for any element of the discrete spectrum with non-negative real-part from Proposition B.2, and a small distance $\delta = 10^{-3}$, since the essential spectrum touches the origin. Note that analytically, the Evans function can be extended up to the origin, see e.g. Faye and Holzer (2020), due to the lack of a discrete eigenvalue zero.

As $d \rightarrow 0$, the computation of the Evans function gets numerically unstable. We get reliable results for $d \ge 0.1$, and found no points of the non-negative discrete spectrum for all choices of *r*, *d* as in Table 1. One of the resulting contours is depicted in Fig. 5, all others were very similar.

## A priori estimates for the left tail of the PDE

We provide the a priori bounds in the regime $x \rightarrow - \infty$ via a Feynman–Kac formula for shifted Brownian motion, which we stop at the origin. The following Lemma C.1 is standard, check e.g. chapter 4.4 in Karatzas and Shreve (1991) for more examples and a profound theoretical treatment. For linear problems (e.g. super-solutions in certain regimes of a PDE), this allows for easy estimates:

### Lemma C.1

For $c, L, M \in \mathbb {R}$, let $u(t,x): \mathbb {R}^+_0 \times \mathbb {R} \rightarrow \mathbb {R}$ be a solution of

C.1 $$\begin{aligned} u_t = \frac{1}{2} \Delta _x u(t,x) + c u_x(t,x) + L u(t,x) + M, \end{aligned}$$

twice differentiable in space and once differentiable in time. For fixed $x_0 < 0$, let $W_t = B_t + ct + x_0$ be a shifted Brownian motion starting in $x_0$. Denote the hitting time $T_0:= \inf \{ t \ge 0: W_t = 0\}$. Assume that for some $t >0$:

C.2 $$\begin{aligned} \sup _{x \le 0} \, \vert u(t=0,x) \vert< \infty \, \text { and } \sup _{s \in [0,t]} \, \vert u(s,x=0) \vert < \infty . \end{aligned}$$

It then holds that

C.3 $$\begin{aligned} \begin{aligned} u(t,x_0)&= \mathbb {E}_{x_0} \Big [ e^{L \cdot t} \cdot u \big ( 0 , W_{t} \big ) ; \, T_0 >t \Big ] \\&\quad + \mathbb {E}_{x_0} \Big [ e^{L \cdot T_0} \cdot u \big ( t- T_0 , 0 \big ); \, T_0 \le t \Big ] \\&\quad + \frac{M}{L} \cdot \mathbb {E}_{x_0} \Big [ e^{L (t \wedge T_0)} \Big ]. \end{aligned} \end{aligned}$$

### Proof

The proof is standard: fix $x_0$ and *t* and apply Itos formula to the stochastic process

C.4 $$\begin{aligned} X_s := e^{L \cdot s} \cdot u(t-s,W_s), \quad s \in [0,t]. \end{aligned}$$

It follows that

C.5 $$\begin{aligned} dX_s&= L e^{L \cdot s} u(t-s,W_s) ds \nonumber \\&\quad + e^{L \cdot s} \cdot \Big [ - u_s ds + u_x \, dBs + u_x c ds + \frac{1}{2} u_{xx} ds \Big ] \nonumber \\&= e^{Ls} \Big [ u_x \, dBs - M ds]. \end{aligned}$$

The last expression consists of a local martingale and a drift, thus

C.6 $$\begin{aligned} u(t,x_0) = X_0 = \mathbb {E}_{x_0}[X_0] = \mathbb {E}_{x_0}[X_{t\wedge T_0}] + M \cdot \mathbb {E}_{x_0} \Big [ \int _0^{t \wedge T_0} e^{Ls} \, ds \Big ], \end{aligned}$$

since $||u||_\infty$ can grow at most exponentially in time. The claim follows by splitting the cases $t < T_0$ and $t \ge T_0$. $\square$

We will need the distribution of the hitting time $T_0$ as in Lemma C.1. It can be shown (e.g. by a Girsanov transformation and the reflection principle), that for $x_0 < 0$, the distribution of $T_0$ has density

C.7 $$\begin{aligned} \mathbb {P}_{x_0} [ T_0 = dt] = \frac{-x_0}{\sqrt{2 \pi t^3}} \cdot e^{ - \frac{(-x_0 - ct)^2}{2t} } \cdot \mathbb {1}_{t > 0} \, dt. \end{aligned}$$

For any speed $c>0$, this defines a probability density, so the hitting time is almost surely finite.

We can now estimate the decay of *A*(*t*, *x*) at the back of the wave. In an analogue fashion, we could also prove that *I*(*t*, *x*) grows at most exponentially in *x*, given that $A(t,x) \le 1$, but we do not need this result.

### Proposition C.2

Let *A*(*t*, *x*), *I*(*t*, *x*) be a non-negative solution of the PDE (1.1) in the moving frame $x = z -ct$, for a speed $c > 0$. Assume that there exist constants $K, \delta , \mu _0 >0$, such that the initial data fulfill

C.8 $$\begin{aligned} I(0, x)&\ge 1 + \delta &  \qquad \forall x \le 0, \nonumber \\ A(0,x)&\le K e^{\mu _0 x} &  \qquad \forall x \le 0, \nonumber \\ A(0,x) + I(0,x)&\le K &  \qquad \forall x \in \mathbb {R}. \end{aligned}$$

Moreover, assume that for some time $t \in (0, \infty ]$, it holds that

C.9 $$\begin{aligned} I(s, x = 0)&\ge 1 + \delta &  \qquad \forall s \in [0,t). \end{aligned}$$

Then, there exist $C, \zeta >0$ that are independent of *t*, such that:

C.10 $$\begin{aligned} \forall s \in [0,t), \, x \le 0: \quad&i)&\quad I(s,x) \ge 1 + \delta , \end{aligned}$$

C.11 $$\begin{aligned}&ii)&\quad A(s,x) \le C e^{\zeta x}. \end{aligned}$$

### Proof

Without loss of generality let $K \ge 1$. By boundedness of the initial data, a solution exists for all times. Since $K \ge 1$, it holds that $A (t,x) \le K$ for all $t \ge 0$.

For the first Bound (C.10), note that the solution *H*(*t*, *x*) of

C.12 $$\begin{aligned} H_t = d \cdot H_{xx} + c \cdot H_z, \quad H(0,x) = I(0,x), \end{aligned}$$

is a sub-solution for *I*(*t*, *x*), which is not affected by any negative reaction-term. We rescale space, introducing $x = \frac{1}{\sqrt{2d}} y$, such that *H* fulfills $H_t = \frac{1}{2} H_{yy} + \frac{c}{\sqrt{2d}} H_y$. We can now apply Lemma C.1 with $L = M = 0$. For all $y_0 \le 0$ and $t \ge 0$, it holds that

C.13 $$\begin{aligned} H(t,y_0 ) = \mathbb {E}_{y_0}[H(0, W_t) ; \, T_0 >t ] + \mathbb {E}_{y_0} [H(t-T_0, 0); \, T_0 \le t] \ge 1 + \delta , \end{aligned}$$

which is nothing but a maximum principle for a diffusion subject to a moving boundary condition.

Now, given that $I(t,x) \ge 1+\delta$ for all $x \le 0$, the solution of

C.14 $$\begin{aligned} J_t = J_{xx} + c \cdot J_x - \delta J \end{aligned}$$

is a super-solution for *A*. Again, we rescale $x = \frac{1}{\sqrt{2}} y$, such that *J* fulfills

C.15 $$\begin{aligned} J_t = \frac{1}{2} J_{yy} + \frac{c}{\sqrt{2}} J_y - \delta J. \end{aligned}$$

We write $\tilde{c} = c / \sqrt{2}$. It holds that for all $y_0 \le 0$:

C.16 $$\begin{aligned} J(t,y_0)&= \mathbb {E}_{y_0} \Big [ e^{-\delta t} \cdot J \big ( 0 , W_{t} \big ) ; \, T_0 >t \Big ] \end{aligned}$$

C.17 $$\begin{aligned}&\quad + \mathbb {E}_{y_0} \Big [ e^{-\delta T_0} \cdot J \big ( t- T_0 , 0 \big ); \, T_0 \le t \Big ] . \end{aligned}$$

We first estimate (C.16). Therefore, we fix $y_0 \le 0$ and differentiate between small and large times. To begin with, we assume that

C.18 $$\begin{aligned} ( 2\tilde{c} + \mu _0 ) t \ge -y_0. \end{aligned}$$

Since $A \le K$, the term (C.16) is bounded by

C.19 $$\begin{aligned} K e^{- \delta t} \le K e^{ \frac{\delta }{2\tilde{c} + \mu _0 } y_0 }. \end{aligned}$$

Next, we consider small times $( 2\tilde{c} + \mu _0 ) t \le -y_0$. The term (C.16) can be bounded by

C.20 $$\begin{aligned}&\mathbb {E}_{y_0} \big [ J(0,W_t) ; T_0 > t \big ] \le \mathbb {E}_{y_0} \big [J(0,W_t) \big ] \le \mathbb {E}_{y_0} \big [ K \cdot e^{ \mu _0 W_t } \big ] \nonumber \\&\quad = K \exp \big ( \mu _0 ( y_0 + \tilde{c}t ) \big ) \cdot \mathbb {E}_0 [ \exp (\mu _0 B_t) ], \end{aligned}$$

where $B_t$ is a standard Brownian motion. Using the moment generating function of $B_t$ and inserting our small time estimate, we get

C.21 $$\begin{aligned}&= K \exp \left( \mu _0 \left[ y_0 + \tilde{c}t + \frac{t \mu _0}{2}\right] \right) \le K \exp \left( \frac{\mu _0}{2} y_0\right) . \end{aligned}$$

We continue with (C.17). For this, we use the distribution of the hitting time $T_0$, see (C.7). In the following, let *C* be a universal constant. We estimate

C.22 $$\begin{aligned} \begin{aligned}&\mathbb {E}_{y_0} \Big [ e^{-\delta T_0} \cdot J \big ( t- T_0 , 0 \big ); \, T_0 \le t \Big ] \le C \mathbb {E}_{y_0} [ e^{-\delta T_0 } ] \\&\quad = \frac{-y_0}{\sqrt{2 \pi }} \int _0 ^\infty \exp (-\delta s) \cdot s^{-3/2} \cdot \exp \left( -\frac{1}{2s} (-y_0 -\tilde{c}s)^2 \right) \, ds. \end{aligned} \end{aligned}$$

Again, we differentiate between small and large times. First, consider

C.23 $$\begin{aligned}&\frac{-y_0}{\sqrt{2 \pi }} \int _{\frac{-y_0}{2 \tilde{c} }} ^\infty \exp (-\delta s) \cdot s^{-3/2} \cdot \exp \left( -\frac{1}{2s} (-y_0 -\tilde{c}s)^2 \right) \, ds \nonumber \\&\quad \le -Cy_0 \cdot \left( \frac{ \vert y_0 \vert }{2 \tilde{c}} \right) ^{-3/2} \int _{\frac{-y_0}{2 \tilde{c}}} ^\infty \exp (-\delta s) \, ds.\end{aligned}$$

For $y_0 \le -1$, this can be bounded by

C.24 $$\begin{aligned}&\le C \exp \left( \frac{\delta }{2 \tilde{c}} y_0 \right) . \end{aligned}$$

We continue with the small time estimate:

C.25 $$\begin{aligned}&\frac{-y_0}{\sqrt{2 \pi }} \int _0^{\frac{-y_0}{2 \tilde{c} }} \exp (-\delta s) \cdot s^{-3/2} \cdot \exp \left( -\frac{1}{2s} (-y_0 -\tilde{c}s)^2 \right) \, ds \nonumber \\&\le -Cy_0 \int _0^{\frac{-y_0}{2 \tilde{c} }} s^{-3/2} \cdot \exp \left( -\frac{y_0^2}{4s} \right) \, ds \nonumber \\&\le -Cy_0 \frac{4}{y_0^2} \int _0^{\frac{-y_0}{2 \tilde{c} }} \sqrt{s} \cdot \frac{y_0^2}{4s^2} \exp \left( - \frac{y_0^2}{4s} \right) \, ds \nonumber \\&\le - \frac{C}{y_0} \sqrt{\frac{-y_0}{2 \tilde{c}}} \cdot \Big \vert _0^{\frac{-y_0}{2 \tilde{c} }} \exp \left( - \frac{y_0^2}{4s} \right) \, ds \end{aligned}$$

For $y_0 \le -1$, this can be bounded by

C.26 $$\begin{aligned}&\le C \exp \left( \frac{\tilde{c}}{2} y_0 \right) \end{aligned}$$

Lastly, remark that for $y \ge -1$, the trivial bound $J(t,y) \le K$ holds, since $A \le K$. Considering the Inequalities (C.19), (C.21), (C.24), (C.26), and re-substituting $\tilde{c} = c / \sqrt{2}$, the upper Bound (C.11) for *A*(*t*, *x*) follows. $\square$

## Geometric singular perturbation theory

For passing continuously from $d = 0$ to $d \ll 1$ in System (1.3), we apply geometric singular perturbation theory for smooth systems, based on work by Fenichel (1979), Jones (1995). We briefly present the underlying theory and its application to the present case. Given two $C^\infty$ functions *f* and *g*, consider the following smooth field:

D1 $$\begin{aligned} \begin{aligned} \frac{d }{dt} z&= f(z,y,d), \\ \frac{d }{ dt} y&= \epsilon g(z,y,d), \quad \epsilon \sim 0. \end{aligned} \end{aligned}$$

The variable *z* is called the *fast variable*, the variable *y* is called the *slow variable*. Geometric singular perturbation theory leads to a result that describes trajectories that are continuous in $\epsilon$ in a neighborhood of $\epsilon = 0$. Introduced by Fénichel in the 70s (Fenichel 1979), this approach has been classical for dealing with singularly perturbed systems, check (Jones 1995). The central assumption is given as follows, it ensures that the fast variables indeed move along fast stable and unstable manifolds:

### Definition D.1

For $z \in \mathbb {R}^n, y \in \mathbb {R}^l$, consider a fixed point $P \in \mathbb {R}^{n+l}$ of a dynamical system as in (D1). It is called a *normally hyperbolic* critical point, if the linearization of the system around *P* has exactly *l* Eigenvalues that lie on the imaginary axis.

For $\epsilon = 0$, the *l* Eigenvalues with zero real-part of the Jacobian at a normally hyperbolic point must correspond to the slow (constant for $\epsilon = 0$) variables. Only the movement in the fast direction remains. In this setting, a separation of time-scales occurs for $\epsilon \ne 0$ sufficiently small, and one can treat the fast and slow variables separately. The following theorem is a combination of Theorems 1–3 in the monograph of Jones (1995), this compact form is due to Rottschäfer and Wayne (2001):

### Theorem D.2

Given a system of type (D1), such that when $\epsilon = 0$, the system of equations has a compact, normally hyperbolic manifold of critical points, $\mathcal {M}_0$, where we assume that $\mathcal {M}_0$ is given as the graph of a $C^\infty$ function $h^0(y)$. Then for every $p \ge 1$, there exists an $\epsilon _0>0$ such that for all $\epsilon \in [0, \epsilon _0)$, there exists a Manifold $\mathcal {M}_\epsilon$:

1. $\mathcal {M}_\epsilon$ is locally invariant under the flow defined by (D1).
2. $\mathcal {M}_\epsilon = \{ (z,y) \vert z = h^\epsilon (y) \}$, for a function $h^\epsilon$ which is $C^p$ in both $\epsilon$ and *y*, and for *y* in some compact set *S*.
3. There are locally stable manifolds $W^s(\mathcal {M}_\epsilon )$ and unstable ones $W^u(\mathcal {M}_\epsilon )$, that lie within $\mathcal {O}(\epsilon )$ of and are diffeomorphic to $W^s(\mathcal {M}_0)$ and $W^u(\mathcal {M}_0)$, respectively. Here, $W^s(\mathcal {M}_0)$ and $W^u(\mathcal {M}_0)$ are the (fast) stable and unstable manifolds of the unperturbed manifold $\mathcal {M}_0$.

We follow the examples in Jones (1995), Rottschäfer and Wayne (2001), and analyze the present system for $d \sim 0$. We bring System (3.5) into slow–fast form as in Eq. (D1), and change the phase-time to the fast phase-time $t:= x / d$:

D2 $$\begin{aligned} \begin{aligned} \frac{d}{ d t } i'&= - [ci' + ra + a(a+i)] =: f(a,a',i,i'), \\ \frac{d}{ d t } \begin{pmatrix} a \\ a' \\ i \end{pmatrix}&= d \cdot \begin{pmatrix} a' \\ a(a+i) -a -ca'\\ i' \end{pmatrix} =: d \cdot g(a,a',i,i'). \end{aligned} \end{aligned}$$

Here $$(a,a',i) $$ are the slow variables and $$i' $$ is the fast variable. For $$d = 0$$, there exists a three-dimensional smooth manifold of critical points

D3 $$\begin{aligned} \mathcal {M}_0 = \Big \{ (a,a',i,i') \in \mathbb {R}^4\Big \vert \, i' = h^0(a,a',i) := - \frac{ a(a+i+r) }{c } \Big \}. \end{aligned}$$

We check normal hyperbolicity of $\mathcal {M}_0$. For $d = 0$, we calculate the Jacobian of the system:

D4 $$\begin{aligned} J_{(a,a',i,i') \vert M_0} = \begin{pmatrix} 0 & \quad 0 & \quad 0 & \quad 0 \\ 0 & \quad 0 & \quad 0 & \quad 0 \\ 0 & \quad 0 & \quad 0 & \quad 0 \\ r + 2a +i & \quad 0 & \quad a & \quad -c \end{pmatrix}, \end{aligned}$$

which has a single non-zero Eigenvalue $\lambda = -c$, with fast direction $i'$. Thus, all points in $\mathcal {M}_0$ have a fast stable manifold of dimension one and no unstable manifold (in fact, $\mathcal {M}_0$ is even a global attractor). In order to apply Theorem D.2, we now choose an arbitrarily large, but bounded subset of $\mathcal {M}_0$. Then, for $d >0$ sufficiently small, there exists a manifold $\mathcal {M}_d$, that is of distance $\mathcal {O}(d)$ to $\mathcal {M}_0$, together with a local unstable manifold $W^s(\mathcal {M}_d)$ that is of distance $\mathcal {O}(d)$ to $W^s(\mathcal {M}_0)$.

For $d \ne 0$, we can undo our transformation and change back to $x= d \cdot t$. Then, we get that on $\mathcal {M}_d$, the perturbed system is close unperturbed system, in the sense that

D5 $$\begin{aligned} \frac{d}{dx} \begin{pmatrix} a \\ a' \\ i \end{pmatrix}&=\begin{pmatrix} a' \\ a(a+i) -a -ca'\\ i' \end{pmatrix} \end{aligned}$$

with $i'=h^d(a,a',i)$, subject to the condition

D6 $$\begin{aligned} ci'&= - a(a+i+r) + \mathcal {O}(d), \end{aligned}$$

which is an $\mathcal {O}(d)$-perturbation of (D3). Equation (D6) implies that the restriction of $S_d$ to $\mathcal {M}_d$ is $\mathcal {O}(d)$-close to the unperturbed system $S_0$. Thus, along $\mathcal {M}_d$, the System $S_d$ can be viewed as a regular perturbation of $S_0$, which we obtain as a (regular) limit as $d \rightarrow 0$:

D7 $$\begin{aligned} \frac{d}{dx} \begin{pmatrix} a \\ a' \\ i \end{pmatrix}&=\begin{pmatrix} a' \\ a(a+i) -a -ca'\\ - \frac{a(a+i+r)}{c} \end{pmatrix}. \end{aligned}$$

We summarize this in

### Corollary D.3

Fix $p \ge 1$ and choose any smooth bounded set $\bar{\mathcal {M}_0} \subset \mathcal {M}_0$, for $\mathcal {M}_0$ as defined in (D3). There exists some $d^*>0$, such that for all $d \in (0, d^*)$, the System (D2) has a three-dimensional $C^p$-manifold $\bar{\mathcal {M}_d}$, invariant under $S_d$ and $\mathcal {O}(d)$-close to $\bar{\mathcal {M}_0}$. The flow on this manifold is an $\mathcal {O}(d)$-perturbation of (D7). Moreover, $\bar{\mathcal {M}_d}$ has no unstable manifold, but only a fast stable manifold $W^s(\bar{\mathcal {M}_d})$ that is locally diffeomorphic to and within range of $\mathcal {O}(d)$ to $W^s(\bar{\mathcal {M}_0})$.

Now let $K >1$ and consider $d>0$ sufficiently small. The fixed point $(a,a',i,i') = (0,0,K,0)$ has an unstable manifold of dimension one (presumably a traveling wave). With the help Corollary D.3, we can track any finite segment of this manifold:

### Corollary D.4

(c.f. Corollary 3.7) Let $K>1, c >0, r \ge 0$. First consider the fixed point $(\bar{a},\bar{a}',\bar{i}) = (0,0,K)$ of $S_0$, together with its one-dimensional unstable manifold $M^-_0(K)$. Fix any semi-open interval $x \in (-\infty , T]$, where *T* is finite, and assume that $M^-_0(K,x)\vert _{x \in (-\infty , T]}$ is smooth and bounded. Lift it into $\mathbb {R}^4$ via (D3).

Now consider the perturbed system $S_d$. There exist some $d^*> 0$ such that for all $d \in (0,d^*)$: the fixed point $(a,a',i,i') = (0,0,K,0)$ has an adjacent one-dimensional unstable manifold $M^-_d(K,x)\vert _{x \in (-\infty , T]}$, that is continuous in *d* and converges to $M^-_0(K,x)\vert _{x \in (-\infty , T]}$ as $d \rightarrow 0$.

### Proof

Fix a finite time-horizon $T \in \mathbb {R}$ such that $M^-_0(K,x)\vert _{x \in (-\infty ,T]}$ is smooth and bounded. Embed it into $\mathbb {R}^4$ by setting $i'=h^0(a,a',i)$, see (D3). Let $\bar{M_0}$ be a smooth bounded subset of $M_0$, sufficiently large such that

D8 $$\begin{aligned} M^-_0(K,x)\vert _{x \in (-\infty ,T]} \subset \bar{M_0}. \end{aligned}$$

There exists an $d^*>0$, such that for all $d \in [0, d^*)$: there exists a $C^p$ manifold $\bar{M_d}$ that is invariant under $S_d$, and the restriction of $S_d$ to $M_d$ is a perturbation of $S_0$ (D7).

Since $(a,a',i,i') = (0,0,K,0)$ is a fixed point, it can not lie on $W^s(\bar{M_d})$, and therefore must lie within $\bar{M_d}$ itself. Similarly, its unstable manifold, given in Corollary 3.4, must lie within $\bar{M_d}$. However, along $\bar{M_d}$, the flow $S_d$ is a regular perturbation of the unperturbed flow $S_0$, with a perturbation of order $\mathcal {O}(d)$. Thus, the finite segment $M^-_0(K,x)\vert _{x \in (-\infty ,T]}$ is continuous in *d*, for $d \in (0, d_1)$, with $d_1$ possibly smaller than $d^*$, by a regular perturbation analogue to Proposition 3.6. $\square$

## Declarations

### Numerical analysis

The numerical analysis of the spectrum in Appendix B was performed via STABLAB (Barker et al. 2015). The simulations of the Reaction–Diffusion System (1.1) were done with Wolfram Mathematica. The code can be accessed upon request.
